# The effect of a therapeutic smartphone application on suicidal ideation in young adults: Findings from a randomized controlled trial in Australia

**DOI:** 10.1371/journal.pmed.1003978

**Published:** 2022-05-31

**Authors:** Michelle Torok, Jin Han, Lauren McGillivray, Quincy Wong, Aliza Werner-Seidler, Bridianne O’Dea, Alison Calear, Helen Christensen

**Affiliations:** 1 University of New South Wales, Sydney, Australia; 2 Western Sydney University, Sydney, Australia; 3 The Australian National University, Canberra, Australia; Stellenbosch University, SOUTH AFRICA

## Abstract

**Background:**

Suicidal ideation is a major risk for a suicide attempt in younger people, such that reducing severity of ideation is an important target for suicide prevention. Smartphone applications present a new opportunity for managing ideation in young adults; however, confirmatory evidence for efficacy from randomized trials is lacking. The objective of this study was to assess whether a therapeutic smartphone application (“LifeBuoy”) was superior to an attention-matched control application at reducing the severity of suicidal ideation.

**Methods and findings:**

In this 2-arm parallel, double-blind, randomized controlled trial, 455 young adults from Australia experiencing recent suicidal ideation and aged 18 to 25 years were randomly assigned in a 2:2 ratio to use a smartphone application for 6 weeks in May 2020, with the final follow-up in October 2020. The primary outcome was change in suicidal ideation symptom severity scores from baseline (T0) to postintervention (T1) and 3-month postintervention follow-up (T2), measured using the Suicidal Ideation Attributes Scale (SIDAS). Secondary outcomes were symptom changes in depression (Patient Health Questionnaire-9, PHQ-9), generalized anxiety (Generalized Anxiety Disorder-7, GAD-7), distress (Distress Questionnaire-5, DQ5), and well-being (Short Warwick–Edinburgh Mental Well-Being Scale, SWEMWBS). This trial was conducted online, using a targeted social media recruitment strategy. The intervention groups were provided with a self-guided smartphone application based on dialectical behavior therapy (DBT; “LifeBuoy”) to improve emotion regulation and distress tolerance. The control group were provided a smartphone application that looked like LifeBuoy (“LifeBuoy-C”), but delivered general (nontherapeutic) information on a range of health and lifestyle topics. Among 228 participants randomized to LifeBuoy, 110 did not complete the final survey; among 227 participants randomized to the control condition, 91 did not complete the final survey. All randomized participants were included in the intent-to-treat analysis for the primary and secondary outcomes. There was a significant time × condition effect for suicidal ideation scores in favor of LifeBuoy at T1 (*p* < 0.001, *d* = 0.45) and T2 (*p* = 0.007, *d* = 0.34). There were no superior intervention effects for LifeBuoy on any secondary mental health outcomes from baseline to T1 or T2 [*p*-values: 0.069 to 0.896]. No serious adverse events (suicide attempts requiring medical care) were reported.

The main limitations of the study are the lack of sample size calculations supporting the study to be powered to detect changes in secondary outcomes and a high attrition rate at T2, which may lead efficacy to be overestimated.

**Conclusions:**

LifeBuoy was associated with superior improvements in suicidal ideation severity, but not secondary mental health outcomes, compared to the control application, LifeBuoy-C. Digital therapeutics may need to be purposefully designed to target a specific health outcome to have efficacy.

**Trial registration:**

Australian New Zealand Clinical Trials Registry ACTRN12619001671156

## Abstract 2

In a randomized controlled trial, Michelle Torok, Jin Han, and colleagues study the effectiveness of the LifeBuoy therapeutic smartphone application for reducing suicidal ideation in young adults in Australia.

## Author summary

**Why was this study done?:**

- Young people are a priority group for suicide prevention efforts, accounting for around one-third of all suicides.
- Suicidal ideation confers a moderate risk for suicide attempt and death, making it a novel, modifiable target for suicide prevention.
- Young people are poor help seekers for suicidal thoughts and behaviors; however, their willingness to engage with technology for mental health support creates enormous potential for smartphone interventions to improve access to help in this group.
- We identified only 2 prior trials of smartphone applications for treating suicidal ideation in younger people, neither which was tested in general population, nonclinical samples. Randomized trials are needed to understand if self-guided digital therapeutics work, are safe, and should be made available to young people.

**What did the researchers do and find?:**

- We conducted a 2-arm parallel randomized controlled trial to examine the effects of a brief, self-guided smartphone intervention (LifeBuoy) designed to reduce the severity of suicidal ideation by teaching emotional regulation and distress tolerance skills compared to a nontherapeutic, attention-matched “sham” application. Participants had access to the application they were randomly assigned to for 6 weeks and then were assessed at the end of the intervention period and 3 month later to determine if their baseline suicidal ideation scores had reduced.
- Young adults who received the LifeBuoy application showed greater reductions in suicidal ideation both immediately and at 3 months after the intervention compared to those who had received the control application. At postintervention, approximately 3 out of 4 young adults in the LifeBuoy group no longer had suicidal ideation score in a threshold that placed them at high risk of suicide attempt, compared to 1 out of 2 in the control group.
- Depression, anxiety, distress, and well-being symptoms improved from baseline to postintervention for young adults in both groups, but the LifeBuoy application did not lead to superior improvements above and beyond those observed in the control group.
- The LifeBuoy application appeared to be safe, with no young adults in the intervention group reporting suicide attempts at final follow up.

**What do these findings mean?:**

- Despite its brief nature and self-guided format, this dialectical behavior therapy (DBT) smartphone application can be used to modestly reduce the severity of suicidal ideation in young adults, making it a potentially useful therapeutic support for young people who might not otherwise be able or willing to access face-to-face treatment.
- Smartphone applications appear to work best when designed to target a specific health problem, with the current study supporting findings from other studies that digital therapeutics appear to be less effective for secondary health problems.
- Further investigation is needed to understand the full clinical meaningfulness of the findings, including which young people might benefit the most from this intervention.

## Introduction

Young people have repeatedly been identified as a group requiring specific attention in suicide prevention efforts. Globally, around one-third of all suicides occur among those aged 15 to 29 years [1], and rates of youth suicide are rising in many countries, including Australia [2], the United Kingdom [3], and the United States [4]. To prevent suicide, it is important to intervene in suicidal behaviors that may signal an increasing risk for suicide death, such as intentional self-harm and suicidal ideation [5]. Suicide attempt and ideation are estimated to occur in around 1% and 11% of community-based young people each year, respectively [6], while suicide mortality occurs in less than 0.02% of all 15 to 24 year olds according to national data registries (e.g., 2). Although pooled findings indicate that the odds of a future suicide attempt are only 1.88 times higher among individuals with suicidal ideation than those without [5], prior evidence shows one-third of youth with suicidal ideation will develop a suicide plan, and 60% with a plan will make a suicide attempt [7]. This risk, considered alongside the prevalence, indicates that suicidal ideation is a novel target for intervention targeting young people.

Increasing trends in youth suicide alongside the substantial and unchanging mental health burden among those aged 0 to 24 years [8] creates considerable uncertainty as to whether there is sufficient availability of treatment services and workforce capacity to meet supply and demand needs over the coming decades. Access to mental health services is already grossly inadequate in regions where suicide rates are the highest, with only 1.6% to 7.2% of those in need accessing such services in low-to middle-income countries, while access rates are 2 to 3 times greater in higher income countries [9]. Similar patterns of higher sucide rates and reduced access to mental health services are echoed in rural and remote regions of high income countries [10,11]. Developing models of treatment that are effective, translatable, and scalable is a salient priority for suicide prevention.

For young people, such services need to capitalize upon how they want to access and engage with help as they are one of the groups least likely to seek help. It is estimated that up to 70% of those aged 12 to 18 years with suicidal thoughts and/or behaviors do not actively utilize mental health services because of embarrassment and stigma, difficulty expressing suicide concerns, lack of support, and a preference for self-reliance [12]. For these reasons, technology-enabled solutions, such as smartphone interventions, may be particularly appropriate models of care for young people. Not only is smartphone ownership and use ubiquitous among those aged 13 to 29 years, but also many (40% or more) report accessing apps specifically for mental well-being [13]. This willingness to engage with technology for mental health support creates enormous potential for smartphone interventions to genuinely address service access gaps.

Adults can readily access and benefit from technology-enabled solutions that directly target suicidal ideation and/or behaviors [14,15]; however, the evidence for this is largely derived from trials of web-based programs rather than smartphone interventions. To our best knowledge, there have only been 2 published studies of brief, unsupervised smartphone interventions designed to treat suicidal ideation and/or behavior in younger adults. In both studies, these interventions were unable to demonstrate superior effects for suicidal ideation [16,17], although one study did show an intervention effect for suicidal plans and suicidal behavior [16]. This latter finding, together with meta-analytic evidence that smartphone applications have a moderate effect on depressive symptoms [18] and a small effect of anxiety symptoms [19], shows promise for these interventions. To advance the field and realize the potential of smartphone applications as effective components of suicide prevention efforts, more trials of interventions that directly target distress processes specific to suicide are needed, rather than more trials of mental health interventions that incidentally measure suicidal outcomes. This need for development and testing of purpose designed treatments is because meta-analyses have shown that interventions directly targeting suicidal behaviors can reduce ideation in the short-term, whereas broader mental health interventions are unable to [14,20].

The current knowledge gaps in the availability of, and evidence for, technology-enabled treatment models specifically for young people informed the development of a targeted self-guided smartphone application (known as LifeBuoy). The LifeBuoy application is primarily based on dialectical behavior therapy (DBT), a specific modified form of cognitive behavior therapy designed to treat persistent emotional dysregulation as a means to preventing self-harm and suicidal behaviors [21]. In DBT treatment, individuals are taught to change unhelpful ways of thinking and behaving, develop healthy distress tolerance skills, and regulate their emotions. To the best of our knowledge, the LifeBuoy application represents the first intervention of its kind to be tested in a randomized trial setting. Since many young people seek help directly online, via their phones [22], we opted to test the intervention as an app available in the app store and promoted the trial via social media. These conditions were likely to mimic the way the intervention would be delivered and used in the community if effectiveness were to be established. The primary objective of this study was to investigate the efficacy of the Lifebuoy smartphone application in reducing the severity of suicidal thoughts when compared with an attention-matched smartphone application (LifeBuoy-C). A secondary objective was to examine these effects for broader mental health outcomes of depression, anxiety, distress, and well-being.

## Methods

This study is reported as per the Consolidated Standards of Reporting Trials (CONSORT) guideline (S1 CONSORT Checklist) [23]. The trial protocol (S1 Protocol) was prospectively registered on the Australian New Zealand Clinical Trials Registry (ACTRN12619001671156) and has also been published elsewhere [24]. The intervention app was registered with the Therapeutic Goods Administration Clinical Trial Notification scheme (CT-2020-CTN-00256-1-v1).

## Study design and participants

This 2-arm parallel, double-blind, randomized controlled trial was conducted at the Black Dog Institute, Australia. Participants were recruited via targeted advertisements on the social media site, Facebook, between May 11, 2020 and May 22, 2020. Eligible participants were between 18 and 25 years of age, in the community (nonclinical sample), residing in Australia at the time of registration, and who responded in the positive to the question “have you experienced suicidal thoughts in the past 12 months?” Eligible individuals also had to own a smartphone (versions Android 5 and iOS 9 or higher) and be fluent in English. Participants were excluded if they had attempted suicide in the 1 month prior to trial registration and/or had a current or lifetime diagnosis of psychosis or bipolar disorder. These exclusion criteria were designed to ensure that we were able to safely manage trial participation. Ineligible participants were provided with phone numbers for appropriate 24-hour crisis support services at the conclusion of the screening and offered the opportunity to speak to the research team’s clinical psychologist. Being in current receipt of other forms of mental health treatment was not an exclusion criterion. The study was approved by the University of New South Wales Human Research Ethics Committee (HC190764) and required written opt-in consent by participants.

### Randomization and masking

Upon completing the baseline survey, participants were randomly assigned (2:2) to the intervention or control group within a block design (4 per block). Randomization was handled via a computer-generated algorithm integrated into the Black Dog Institute’s bespoke trial management software (Research Engine), which facilitates allocation concealment. The randomization outcome was communicated to participants by the receipt of a link via email or SMS (dependent on the participant’s preferred mode of contact) to 1 of 2 apps with no research personnel directly involved in the intervention delivery. All study chief investigators and participants were masked to intervention assignment until completion of the final survey.

### Procedures

The trial was run online using the Research Engine, which managed consent, screening, registration, randomization, data collection, and intervention delivery. The Facebook advertisements directed participants to an online trial portal, where they completed individual consent and eligibility screening. To proceed, eligible participants had to provide an email address and mobile phone number and create a unique password so that they could access surveys for the duration of the trial and receive links to their allocated application and reimbursements for survey completion. Participants were asked to complete surveys at baseline [T0; day 0], postintervention [T1; day 43], and 3-month postintervention [T2; day 132]). At T1 and T2, participants received an electronic link to a $20 e-voucher if they completed the surveys. Participants were sent a link to their allocated application following the baseline survey and had 7 days to download it from the app store. Each condition had 6 weeks of access to their allocated intervention. Participants had 7 days to complete each survey, receiving 2 reminder communications before the survey link was deactivated.

### Intervention and control arms

Participants in the intervention condition received a brief, 7-module, self-guided DBT smartphone application (“LifeBuoy”) designed to improve emotional regulation and increase distress tolerance skills. DBT is one of the most effective therapeutic approaches for reducing suicidal thoughts and suicide attempts in young people [25]. The frequency of the modules was flexible; however, participants needed to complete one module to unlock the next. Participants were able to return to modules as often as desired, with each estimated to approximately 5 minutes to complete. One DBT skill was introduced and practiced per module, with brief education content provided at the start of each module to explain the skill, followed by an interactive exercise or feature to practice the skill, such as quizzes, a brief animated breathing tool, and audio files for guided mindfulness and self-soothing. All interactive features were built into the application for ease of access. Following the exercise component of the module, participants were presented with a brief overview of the importance of that skill in the context of managing suicidal thoughts and tips as to the frequency with which the skill should be practiced before exiting the module. The skills taught were self-soothing, pros and cons, distress tolerance, Activities, Contributing, Comparisons, Emotions, Push away, Thoughts, and Sensation (ACCEPTS), and radical acceptance. The ACCEPTS skill was delivered across 2 modules. The final module focused on values and goal setting, providing a behavioral activation strategy to encourage participants to engage in activities that enhance their sense of purpose, pleasure, and provide a sense of mastery of the skills taught in the preceding modules. In this module, participants were asked to identify the top 3 values important to them (out of a predefined list of 10) and then set a goal against each one to achieve over the course of the 6 weeks. Weekly reminders were sent to encourage participants to achieve their goals.

The application also contained a toolbox function that provided access to additional distress tolerance activities (i.e., Temperature, Intense exercise, Paced breathing, Paired muscle relaxation (TIPP)) and built-in distraction tools (e.g., a popping bubbles game or a fun quiz) as well as a mood tracker. The modules were presented as islands on a map (S1 Fig), and progress was signaled by the islands changing from being whited out to technicolor. LifeBuoy was developed by researchers at the Black Dog Institute with the involvement of young people with a lived experience of suicide (via focus groups and surveys), to understand and accommodate the perspectives of those who the intervention is intended for.

For our control condition, we created a sham app, LifeBuoy-C, which was designed to match LifeBuoy on expectancy and time on task to control for digital placebo effects. LifeBuoy-C retained the module structure and visual appearance of LifeBuoy, presenting the user with 7 sequential islands (modules). Each module presented educational content on topics peripherally related to mental health, but not derived from a therapeutic model: performance stress, general stress, confidence, value of trying something new, how to achieve goals, the benefits of being present, and the value of being out in nature. Links to short TedX videos were provided on the topic in addition to the educational content to provide an interactive element, and each module was not expected to take more than 5 minutes to complete. Control condition participants who completed the T2 survey were informed of their allocation and provided a link to LifeBuoy with 6 weeks of access enabled.

To support participant safety during the trial, we built in a linked directory of major Australian crisis helplines (e.g., Lifeline and Suicide Call Back Service) into each application, along with a “help” button that, if pressed, sent an email to the research team that the participant wanted to be contacted by the clinical psychologist within the next 24 business hours. Only 2 participants used this latter functionality during the trial. At each survey time point, if participants exceeded cutoff scores of 21 or greater on the Suicidal Ideation Attributes Scale (SIDAS) [26], the research team was alerted via email. In tandem, the participant was sent an alert flagging their score and asking if they wanted to be contacted by the team’s clinical psychologist. If the participant returned a “yes” response, they were contacted by phone within 72 hours. In total, 265 participants at T0, 98 participants at T1, and 65 participants at T2 were sent these email alerts; however, only 5 participants opted to speak with the psychologist.

### Outcomes

The primary outcome measure was the mean change in the total SIDAS score from baseline to T1 and from baseline to T2. The SIDAS is a validated, 5 item, 11-point scale measure (Cronbach’s α = 0.62), which assesses the frequency of ideation (how often have you had thoughts about suicide?), controllability (how much control have you had over these thoughts?), severity (how close have you come to making a suicide attempt?), and impact (to what extent have you felt tormented by thoughts about suicide?, how much have thoughts about suicide interfered with your ability to carry out daily activities?). Total scores range from 0 to 50, and higher scores indicating greater symptom severity. Scores of ≥21 indicate high risk for suicidal behavior.

Secondary efficacy endpoints were between-group comparisons of baseline to postintervention change in scores on the following mental health and well-being measures. Recent depression symptoms (in past 2 weeks) were measured by the Patient Health Questionnaire-9 (PHQ-9; Kroenke, Spitzer [27]), a reliable and valid 9-item measure (Cronbach’s α = 0.85). The PHQ-9 has a total score range of 0 to 27, and ranges of 10 to 14, 15 to 19, and 20 to 27 can be respectively interpreted as moderate, moderate-severe, and severe depression. Anxiety symptoms were measured using the Generalized Anxiety Disorder-7 (GAD-7) [28], which also demonstrated good internal consistency (Cronbach’s α = 0.87). Scores on the GAD-7 range from 0 to 21, with cut points of 5, 10, and 15 being interpreted as mild, moderate, and severe levels of anxiety, respectively. Well-being was measured using the 7-item Short Warwick–Edinburgh Mental Well-Being Scale [SWEMWBS] (score range 7 to 35) [29]. Total scores were transformed (as per set out in [29]) to facilitate the use of parametric statistical analyses. Higher scores represent greater well-being (Cronbach’s α = 0.78). Psychological distress was assessed using the Distress Questionnaire-5 (DQ5) [30]. Total scores range from 5 to 25, and scores of 14 or higher indicate the possibility of a mental health condition [30]. The DQ5 demonstrated good internal consistency (Cronbach’s α = 0.74).

Harms were assessed by asking participants if they had previously ever attempted suicide (at T0 only) and, at all time points, whether they had attempted suicide in the past 30 days (“No, never”, “Yes, once,” and “Yes, more than once”). Participants who reported having a recent suicide attempt were asked whether intervention was required [No care needed, some care needed, required medical care). Events requiring medical care constituted a serious adverse event. The questions assessing harm were adapted from a previous trial of a digital intervention for suicidal ideation [31] All outcomes were assessed via the online surveys and were not locally integrated into the LifeBuoy application.

Details about application download and module completion were automatically recorded by the smartphone application and uploaded to central servers when the devices were connected to internet.

All secondary measures were assessed at baseline (T0), postintervention (T1), and 3-month postintervention follow up (T2).

### Statistical analyses

A prespecified analysis plan outlined in our protocol governed our analyses [24; S1 Protocol], unless identified as post hoc. Power analyses determined that a sample size of 189 participants per intervention group would be sufficient to detect an effect size of 0.30 [14] for our primary outcome at postintervention with at least 80% power and an α criterion of 0.05.

Initial analyses examined differences between conditions at T0. Primary analyses that followed were undertaken on an intent-to-treat basis, including all participants as randomized, regardless of treatment received or withdrawal from the study. To this end, linear mixed models for repeated measures analyses were used to examine primary (SIDAS) and secondary outcomes (PHQ-9, GAD-7, DQ5, and SWEMWBS) across T0, T1, and T2, including all available data from participants. To allow different slopes for the T0 to T1 (intervention) and T1 to T2 (follow-up) periods in models, we used a piecewise approach and specified 2 time variables reflecting these periods, along with Condition (LifeBuoy, LifeBuoy-C), and time × condition interaction terms as fixed effects. An unstructured covariance matrix was employed, and degrees of freedom were estimated using Satterthwaite’s method. Tests for a specific treatment arm or at a specific time point were conducted by recoding relevant variables representing Time and Condition within models. Effect sizes (Cohen’s *d*) were calculated based on modeled mean differences and SDs at the relevant time points.

Trial safety data arr reported as proportion of participants flagged as at risk based on SIDAS scores of ≥21. A generalized linear mixed model analysis with binomial distribution, a logit link, a scaled identity covariance matrix, and degrees of freedom estimated using Satterthwaite’s method was conducted post hoc to model the odds of participants flagged as at risk over time. We reported the odds ratios (ORs) comparing the 2 conditions at each time point. The number of participants who had attempted suicide in the past 30 days at T1 and T2 (including the number requiring medical care) were reported descriptively as part of the safety analyses.

Attrition analyses were used to identify any significant differences in the baseline characteristics between those who completed versus did not complete the T1 and T2 surveys. Adjusted linear mixed models were then run for the primary outcome endpoint (SIDAS) to control for any characteristics found to significantly differentiate completers from noncompleters. For all analyses, *p*-values ≤ 0·050 were considered significant. The software package SPSS Statistics 25.0 was used for all analyses.

### Deviation from study protocol

In the protocol submitted to the Australian New Zealand Clinical Trials Registry (ACTRN12619001671156), the final assessment was described as occurring 4-month postintervention. Due to delays to the development of the LifeBuoy application, the final assessment was changed to 3-month postintervention to ensure the trial would complete in the funding timeline. This change was approved at March 14, 2020, prior to the start of recruitment, by the University of New South Wales Human Research Ethics Committee and is accurately reflected in our published protocol [24].

## Results

**Fig 1:**
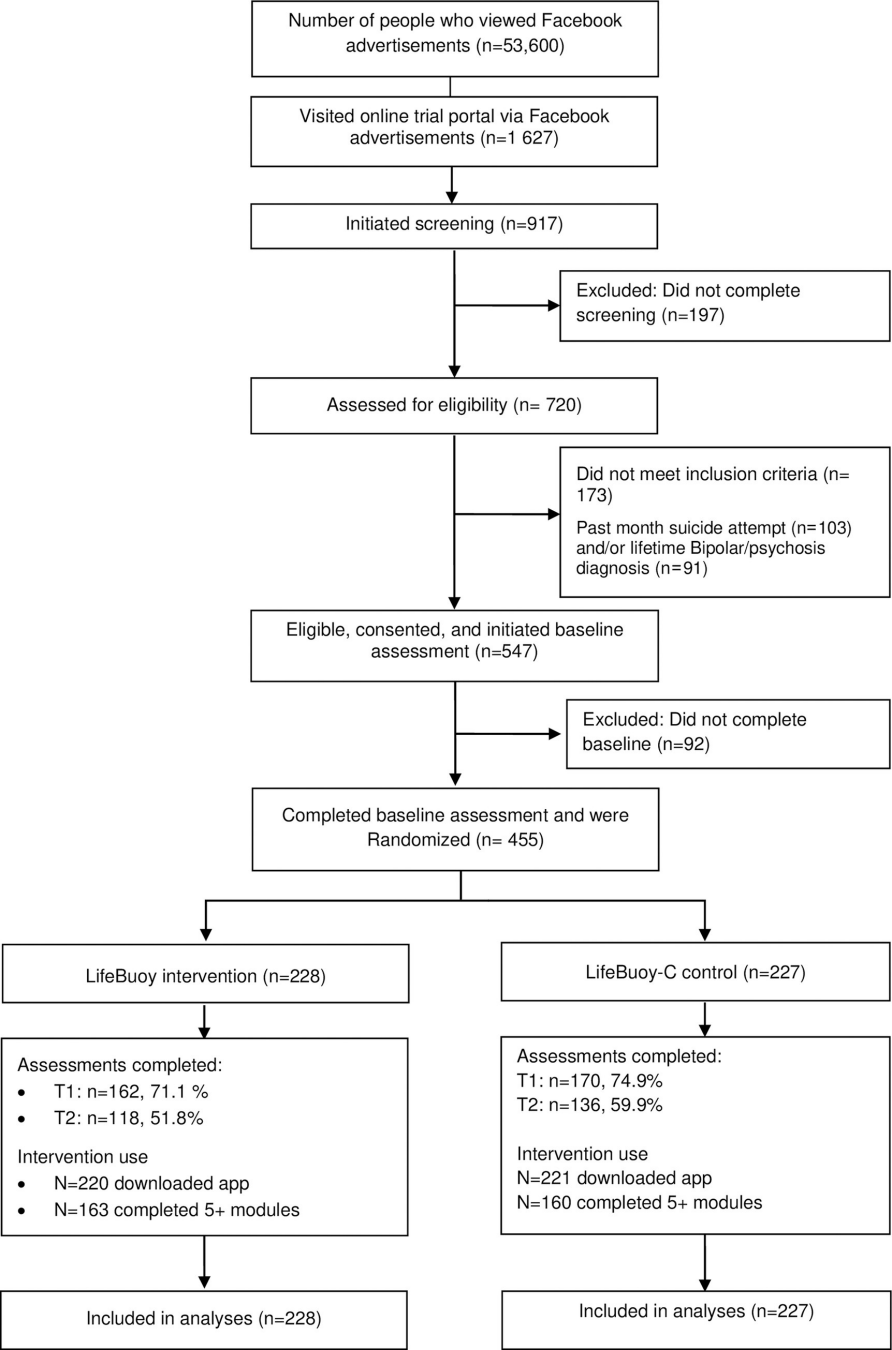
CONSORT trial flow. CONSORT, Consolidated Standards of Reporting Trials.

Fig 1 shows the trial profile. Over 12 days, starting May 11, 2020, 455 participants were randomly assigned to receive LifeBuoy (*N* = 228) or LifeBuoy-C (*N* = 227). The final survey was administered on October 1, 2020. Of these, 332 (73.0%) participants completed (or partially completed) T1 survey, and 254 (55.8%) completed the T2 survey (Fig 1). There were no significant differences in attrition from assessment between the conditions at T1 (Fisher exact test *p* = 0.40) and T2 (Fisher exact test *p* = 0.09) (Fig 1). No participants formally withdrew from the study.

The participant sample was primarily female (*n* = 384, 84.4%) with a mean age of 21.5 years (SD: 2.18). Nearly one-third had completed a tertiary qualification (*n* = 135, 29.7%). Mental health issues were prevalent, with 88.6% (*n* = 403) having ever received a mental health diagnosis and 88.1% (*n* = 401) have ever received mental health treatment. At baseline, gender, age, and other demographic characteristics were similar between conditions (Table 1). For the total sample, the baseline mean SIDAS scores were in the range that indicates high risk of suicidal behavior (≥21; M = 22.61; SD = 8.18). The mean PHQ-9 (M = 17.15; SD = 5.64), GAD-7 (M = 12.29; SD = 5.07), DQ5 (M = 17.87; SD = 3.42), and SWEMWBS (M = 17.15, SD = 2.53) scores were also within clinically relevant ranges, indicating moderate-severe manifestations of these symptoms at baseline.

**Table 1:** Baseline characteristics of the total sample by condition.

	Total sample (*n* = 455)	Control (LifeBuoy-C) (*n* = 227)	Intervention (LifeBuoy) (*n* = 228)
Female (*n*, %)	384 (84.6)	194 (85.5)	190 (83.3)
Age (M, SD)	21.5 (2.18)	21.66 (2.17)	21.37 (2.19)
LGBQI sexual minority, yes (*n*, %)	244 (53.6)	120 (52.9)	124 (54.4)
Education (*n*, %)			
Year 12 (including equivalent) or less	207 (45.5)	99 (44.0)	108 (47.8)
Graduate certificate or diploma	109 (24.0)	61 (27.1)	48 (21.2)
University degree	135 (29.7)	65 (28.9)	70 (31.0)
Living with parents (*n*, %)	246 (54.1)	119 (52.7)	127 (55.9)
Live in metropolitan area (*n*, %)	369 (81.1)	186 (82.3)	183 (80.3)
Not currently working/in paid employment (*n*, %)	168 (36.9)	90 (39.8%)	78 (34.5%)
Ever diagnosed with a mental illness, yes (n %)	404 (88.8)	199 (88.1%)	204 (89.5%)
Have ever received mental health treatment, yes (*n*, %)	402 (88.4)	199 (87.7%)	203 (89.4%)
Lifetime suicide attempt, yes (*n*, %)^a^	199 (43.7)	104 (45.8)	95 (41.7)

LGBQI, Lesbian, Gay, Bisexual, Queer, and Intersex; M, mean; *n*, number; SD, standard deviation.

### Engagement

In total, 437 (96%) participants downloaded the app they were randomized to, with no significant differences between the LifeBuoy (*n* = 220, 96.5%) and control (*n* = 217, 95.6%) conditions (χ^2^(1) = 0.24, Fisher exact *p* = 0.64). There were also no condition differences in the mean number of modules completed (LifeBuoy: M = 6.84; SD = 4.30 versus Control: M = 6.70; SD = 4.8, t[453] = −0.32, *p* = 0.75) nor in the proportion who completed 5 or more modules (defined as “completers”) (LifeBuoy: *n* = 163, 71.5% versus control: *n* = 160, 70.5%; χ^2^(1) = 0.06, Fisher exact *p* = 0.84). There were differences in the median time spent in the application, with the LifeBuoy condition interacting with their application for a significantly greater amount of time overall (LifeBuoy: Median = 1,854.50 seconds/30.91 minutes versus Control: Median = 901.00 seconds/15.02 minutes; Z = −4.43, *p* < 0.001).

### Primary outcome

**Fig 2:**
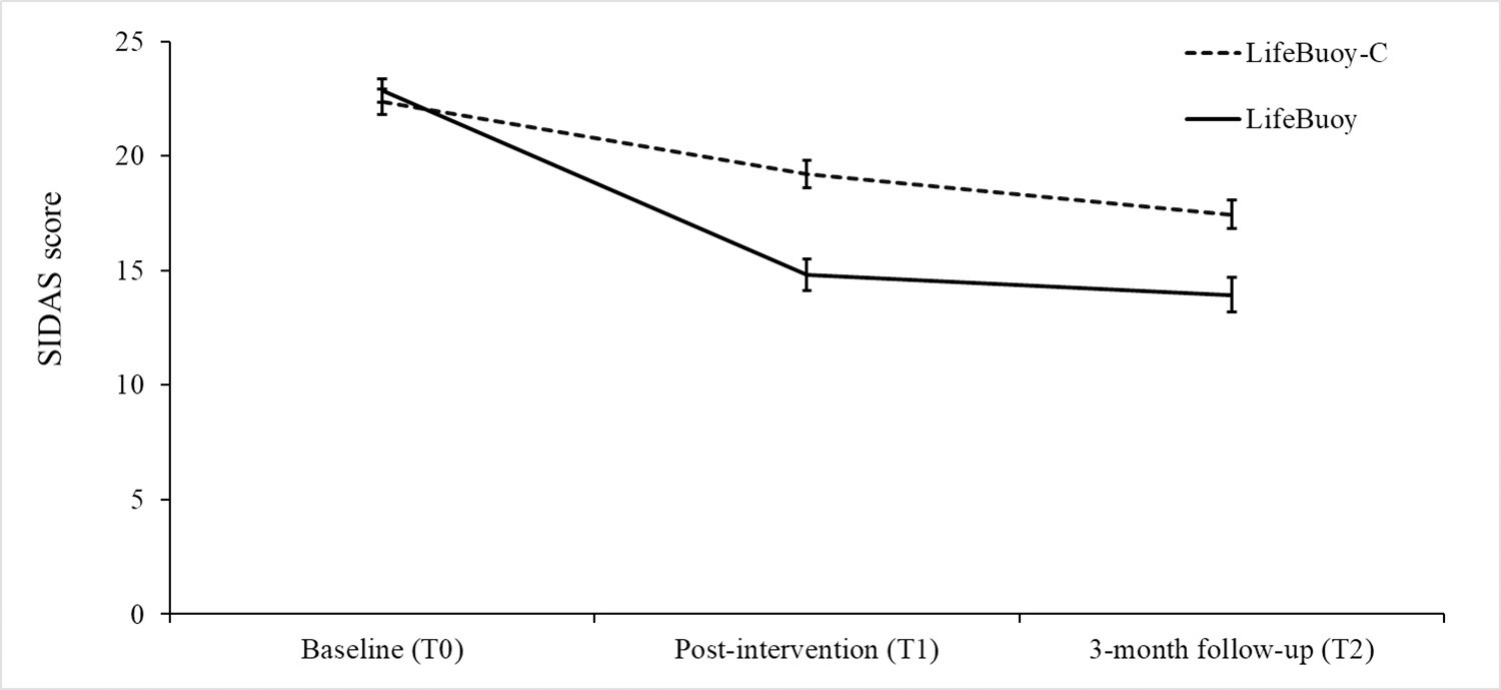
Comparison of SIDAS modeled mean scores at T0, T1, and T2. SIDAS, Suicidal Ideation Attributes Scale; T0, baseline; T1, postintervention; T2, 3-month postintervention.

There was a greater reduction in SIDAS scores from T0 to T1 for LifeBuoy (*B* = −8.05, 95% CI [−9.92, −6.18], t[452.90] = −8.45, p < 0.001, *d* = −1.02) compared to the control condition (*B* = −3.14, 95% CI [−5.04, −1.23], t[463.03] = −3.24, *p* = 0.001, *d* = −0.37; see Table 2, Fig 2), resulting in significantly lower SIDAS scores at T1 (*B* = −4.43, 95% CI [−6.70, −2.16], t[413.83] = −3.83, *p* < 0.001, *d* = 0.45) for those receiving the LifeBuoy intervention. While there was no further significant change in SIDAS scores that occurred from T1 to T2 for the LifeBuoy condition (*B* = −0.86, 95% CI [−2.56, 0.84], t[218.46] = −1.00, *p* = 0.319, *d* = −0.08), T1 to T2 SIDAS scores significantly decreased for the control condition (B = −1.78, 95% CI [−3.53, −0.02], t[226.05] = −2.00, *p* = 0.047, *d* = −0.20). The significantly lower SIDAS scores of the LifeBuoy condition relative to the control condition observed at T1 remained at T2 (B = −3.51, 95% CI [−6.08, −0.94], t[473.81] = −2.69, *p* = 0.007, *d* = 0.34). Over the whole T0 to T2 period, a significant time × condition interaction was also found (see Table 2), with an overall greater reduction in SIDAS scores for LifeBuoy (*B* = −8.91, 95% CI [−10.97, −6.85], t[530.68] = −8.50, *p* < 0.001, *d* = −1.13) versus the control condition (*B* = −4.91, 95% CI [−6.99, −2.83], t[531.94] = −4.64, *p* < 0.001, *d* = −0.58).

**Table 2:** Mean scores, tests of time × condition interactions, and comparisons between time points and conditions.

	LifeBuoy-C				LifeBuoy						
	T0 (M, SD)	T1 (M, SD)	T2 (M, SD)	Changes between time points^a^	T0 (M, SD)	T1 (M, SD)	T2 (M, SD)	Changes between time points^a^	Difference between arms at T1^a^	Difference between arms at T2^a^	Time × condition interactions
SIDAS	22.37 (8.49)	19.25 (9.07)	17.95 (9.25)	ΔT0 to T1 ***p* = 0.001** ΔT1 to T2 ***p* = 0.047** ΔT0 to T2 ***p* < 0.001**	22.85 (7.86)	14.95 (10.4)	14.61 (11.23)	ΔT0 to T1 ***p* < 0.001** ΔT1 to T2 *p* = 0.319 ΔT0 to T2 ***p* < 0.001**	***p* < 0.001**	***p* = 0.007**	T0 to T1: *B* = −4.91, 95% CI [−7.58, −2.24], *t*(458.07) = −3.62, ***p* < 0.001** T1 to T2: *B* = 0.91, 95% CI [−1.53, 3.35], *t*(222.30) = 0.74, *p* = 0.461 T0 to T2: *B* = −4.00, 95% CI [−6.92, −1.07], *t*(531.32) = −2.68, ***p* = 0.008**
PHQ-9	17.24 (5.65)	14.18 (6.40)	13.99 (6.19)	ΔT0 to T1 ***p* < 0.001** ΔT1 to T2 *p* = 0.608 ΔT0 to T2 ***p* < 0.001**	17.07 (5.64)	12.98 (6.44)	14.13 (6.08)	ΔT0 to T1 ***p* < 0.001** ΔT1 to T2 *p* = 0.021 ΔT0 to T2 ***p* < 0.001**	*p* = 0.100	*p* = 0.739	T0 to T1: *B* = −0.94, 95% CI [−2.16, 0.29], *t*(477.64) = −1.50, *p* = 0.135 T1 to T2: *B* = 1.34, 95% CI [0.05, 2.63], *t*(259.23) = 2.04, ***p* = 0.043** T0 to T2: *B* = 0.40, 95% CI [−0.97, 1.78], *t*(567.24) = 0.58, *p* = 0.565
GAD-7	12.00 (5.17)	10.49 (5.39)	10.68 (5.37)	ΔT0 to T1 ***p* < 0.001** ΔT1 to T2 *p* = 0.386 ΔT0 to T2 ***p = 0*.002**	12.57 (4.96)	9.79 (4.90)	10.11 (5.24)	ΔT0 to T1 *p <* 0.001 ΔT1 to T2 *p* = 0.327 ΔT0 to T2 ***p* < 0.001**	*p* = 0.507	*p* = 0.629	T0 to T1: *B* = −0.92, 95% CI [−1.91, 0.07], *t*(519.26) = −1.82, *p* = 0.069 T1 to T2: *B* = 0.07, 95% CI [−1.04, 1.19], *t*(276.96) = 0.13, *p* = 0.896 T0 to T2: *B* = −0.85, 95% CI [−1.95, 0.26], *t*(583.20) = −1.51, *p* = 0.133
DQ5	17.93 (3.54)	15.99 (4.23)	16.05 (3.79)	ΔT0 to T1 ***p* < 0.001** ΔT1 to T2 *p* = 0.832 ΔT0 to T2 ***p* < 0.001**	17.82 (3.30)	15.89 (3.94)	15.98 (3.88)	ΔT0 to T1 ***p* < 0.001** ΔT1 to T2 *p* = 0.663 ΔT0 to T2 ***p* < 0.001**	*p* = 0.940	*p* = 0.929	T0 to T1: *B* = 0.10, 95% CI [−0.71, 0.90], *t*(489.12) = 0.23, *p* = 0.815 T1 to T2: *B* = 0.07, 95% CI [−0.77, 0.92], *t*(457.87) = 0.17, *p* = 0.864 T0 to T2: *B* = 0.17, 95% CI [−0.70, 1.04], *t*(529.44) = 0.38, *p* = 0.702
SWEMWBS	17.20 (2.30)	18.33 (2.82)	18.45 (3.07)	ΔT0 to T1 ***p* < 0.001** ΔT1 to T2 *p* = 0.593 ΔT0 to T2 ***p* < 0.001**	17.09 (2.75)	18.47 (3.13)	18.23 (2.98)	ΔT0 to T1 ***p* < 0.001** ΔT1 to T2 *p* = 0.190 ΔT0 to T2 ***p* < 0.001**	*p* = 0.664	*p* = 0.382	T0 to T1: *B* = 0.27, 95% CI [−0.35, 0.88], *t*(466.49) = 0.85, *p* = 0.395 T1 to T2: *B* = −0.45, 95% CI [−1.12, 0.22], *t*(276.79) = −1.33, *p* = 0.186 T0 to T2: *B* = −0.19, 95% CI [−0.85, 0.48], *t*(520.68) = −0.55, *p* = 0.584

^a^Tests for a specific treatment arm or at a specific time point were conducted by recoding relevant variables representing time and condition within models.

DQ5, Distress Questionnaire-5; GAD-7, Generalized Anxiety Disorder-7; M, mean; PHQ-9, Patient Health Questionnaire-9; SD, standard deviation; SIDAS, Suicidal Ideation Attributes Scale; SWEMWBS, Short Warwick–Edinburgh Mental Well-Being Scale; T0, baseline; T1, postintervention; T2, 3-month postintervention.

To explore the clinical significance of change in suicidal ideation, we undertook an exploratory analysis of item 5 of the SIDAS that measures life interference from suicidal ideation (“how much have thoughts about suicide interfered with the ability to carry out daily activities, such as work, household tasks or social activities”). There was a significant reduction in life interference from suicidal ideation for LifeBuoy compared to the control condition from T0 to T1, leading to lower levels of life interference from suicidal ideation for LifeBuoy at T1 but this was not maintained at T2 (S1 Text).

### Secondary outcomes

Although both conditions improved from T0 to T1 for all secondary outcomes (all |ts| > 5.76, all ps < .001) no differential time × condition effects were evident (see Table 2). At T1 to T2, however, PHQ-9 scores significantly increased in the LifeBuoy condition (B = 1.11, 95% CI [0.17, 2.04], t[260.07] = 2.32, *p* = 0.021, *d* = 0.17) but not the control condition (B = −0.23, 95% CI [−1.12, 0.66], t[258.30] = −0.51, *p* = 0.608, *d* = −0.04).

### Harms

Participants who scored ≥21 on the SIDAS were defined as at high risk for suicidal behavior [23]. At the T1 and T2 surveys, participants in the control condition had significantly greater odds of being flagged as high risk on the SIDAS compared to the intervention condition. Correspondingly, the number needed to treat (NNT) to obtain one participant scoring <21 on the SIDAS was 5 and 8 at the T1 and T2 time points, respectively (Table 3). At the T1 survey, *n* = 4 participants (*n* = 2 in each condition) self-reported having attempted suicide in the past 30 days, while at the T2 survey, 5 participants had attempted suicide in the past 30 days, all in the control condition. Of these 9 suicide attempts, none required medical care.

**Table 3:** Proportion of participants at high risk for suicidal behavior at T0, T1, and T2.

	SIDAS scores ≥ 21 (high risk) *N* (%)		OR^a^ [95% CI]	t	*p*	NNT^d^ [95% CI]
	**LifeBuoy (*n*, %)**	**LifeBuoy-C (*n*, %)**				
T0	136 (59.6)	129 (57.1)	0.97 [0.89, 1.07]	t(930) = −0.55	0.58	-
T1^b^	36 (24.7)	62 (44.9)	1.22 [1.10, 1.37]	t(930) = 3.59	<0.001	5 [4, 11]
T2^c^	26 (26.0)	39 (39.8)	1.14 [1.00, 1.30]	t(930) = 2.01	0.04	8 [4, 119]

^a^OR reflects odds of being flagged as at risk for the LifeBuoy-C condition relative to the LifeBuoy condition. The generalized linear mixed model used to generate the ORs incorporated all participants with at least 1 data point.

^b^Number of participants who completed T1 SIDAS assessment *n* = 284 (*n* = 138 control, 146 intervention).

^c^Number of participants who completed T2 SIDAS assessment *n* = 198 (*n* = 98 control, 100 intervention).

^d^NNT: the number of participants that need to be treated to obtain one participant scoring < 21 on the SIDAS.

OR, odds ratio; NNT, number needed to treat; SIDAS, Suicidal Ideation Attributes Scale; T0, baseline; T1, postintervention; T2, 3-month postintervention.

### Attrition from assessment

In the attrition analysis, those who completed the T1 survey had significantly lower baseline anxiety symptoms (t[445] = 2.3; *p* = 0.02; S1 Table) compared to participants who did not complete it. Compared to participants who did not complete the T2 survey, those who completed it were more likely to have a university degree (χ^2^(1) = 6.60, *p* = 0.10), significantly less baseline depression (t[451] = 2.6; *p* = 0.01), anxiety (t[445] = 2.7; *p* = 0.008), distress (t[444] = 2.3; *p* = 0.02), and greater baseline well-being (t[438] = −2.6; *p* = 0.009; S1 Table). No other significant differences were found. The significant time × condition interactions and significant differences between conditions at T1 and T2 found in the primary outcome analyses of the SIDAS remained after repeating these analyses with adjustment for the variables differentially associated with attrition at T1 and T2 (see Table 4).

**Table 4:** Effect of LifeBuoy relative to LifeBuoy-C on the SIDAS after adjustment for baseline variables associated with attrition.

	Difference between arms at T1^a^^,^^b^^,^^c^	Difference between arms at T2^a^^,^^b^^,^^c^	Time × condition interactions^b^
SIDAS	***p* < 0.001**	***p* = 0.011**	T0 to T1: B = −4.98, 95% CI [−7.66, −2.29], t[450.34] = −3.65, ***p* < 0.001** T1 to T2: *B* = 1.28, 95% CI [−1.21, 3.77], *t*(215.63) = 1.01, *p* = 0.312 T0 to T2: B = −3.70, 95% CI [−6.64, −0.75], t[519.54] = −2.47, ***p* = 0.014**

^a^Tests for a specific time point were conducted by recoding relevant variables representing time within models.

^b^Adjusting for variables differentially associated with attrition at T1 and T2: university degree, depression, anxiety, distress, and well-being at baseline.

^c^In these adjusted analyses, *d* = 0.47 at T1 and *d* = 0.32 at T2 both favoring LifeBuoy (i.e., lower SIDAS scores for those who received the LifeBuoy intervention versus LifeBuoy-C). By comparison, in the original unadjusted analyses, *d* = 0.45 at T1 and *d* = 0.34 at T2 both favoring LifeBuoy.

SIDAS, Suicidal Ideation Attributes Scale; T0, baseline; T1, postintervention; T2, 3-month postintervention.

## Discussion

This study evaluated the efficacy of a brief, unsupervised therapeutic smartphone application for young adults in the community experiencing suicidal ideation. Results show moderate intervention effects for in the immediate postintervention period (*d* = 0.45) and smaller benefits at the 3-month follow up (*d* = 0.34) in reducing the primary outcome, suicidal ideation. Although suicidal ideation reduced in both the intervention and the attention-matched control conditions over time, these reductions were superior in the LifeBuoy condition. For secondary outcomes relating to depression, generalized anxiety, distress, and well-being symptom changes in the expected direction were observed over time, with the exception that depression symptoms increased between the immediate postintervention and 3-month follow up period in the LifeBuoy condition. No significant between-condition effects were reported for any of these secondary outcomes at any time point.

Our results are consistent with pooled meta-analytic findings that digital therapeutics appear to be effective in reducing suicidal ideation [14] yet deviate from the effects reported in standalone trials. That is, prior to this study, 2 brief, unsupervised smartphone applications were tested in 4 unique RCTs involving similarly aged participants with suicidal thoughts or behaviors [16,17], neither application demonstrated an intervention effect for suicidal ideation (*p*s: 0.12 to 0.30). In considering why our findings may differ from previous studies, there is evidence that treatment approach may be an important moderator of efficacy [14,20]. The therapeutic models tested in these previous trials differed from that used in Lifebuoy; one application delivered acceptance and commitment therapy [17] and the other therapeutic evaluative conditioning [16]. Our findings extend prior research showing that DBT can be effectively digitized to treat suicidal ideation [14] by showing promise for this approach in young adults. It is also noteworthy that the immediate effect of Lifebuoy on suicidal ideation in our postintervention period (*d* = 0.45) is comparable to the pooled effects reported for the intensive face-to-face DBT treatment of suicidal ideation (e.g., *d* = 0.48) [32]. When delivered in its face-to-face modality, DBT is a lengthy (4 to 6 months duration) multicomponent program involving individual psychotherapy, multifamily group skills training, coaching, and weekly therapist team consultation [33]. While the intensity of face-to-face DBT is well aligned to its core purpose, i.e., to reduce intentional self-harm behaviors in those with complex mental health issues (e.g., borderline personality and bipolar disorders) [34], our findings suggest that this therapeutic approach be used for the short-term management of suicidal ideation for a less complex cohort of young adult ideators.

The absence of an intervention effect for secondary mental health and well-being outcomes was unexpected yet appears to corroborate findings from prior meta-analytic studies that even face-to-face DBT does not significantly reduce depressive symptoms among adolescents (*p* = 0.099; [32]) nor adults (*p* = 0.08; [33]). Such findings imply that effective therapeutic approaches for the treatment of suicidal ideation may not parallel those used to successfully treat common mental health conditions (e.g., depression and generalized anxiety disorder). Certainly, while cognitive behavioral therapy (CBT) has been successfully translated into digital interventions for depression and anxiety [35], it appears to have little impact on ideation or other suicidal outcomes in comparison to alternative therapies such as DBT [14]. Variation in the clinical usefulness of different treatment approaches substantiates a need to purposefully design digital interventions to a specific health outcome—particularly unsupervised digital therapeutics that are susceptible to being used as multipurpose solutions, potentially to little effect.

Our trial also offered several novel strengths. First, LifeBuoy was codesigned with young people who had a lived experience of suicide to ensure the resulting application aligned to the preferences and needs of end users. Issues of relevance, acceptability, and perceived benefits of digital therapeutics can be addressed through codesign and may help overcome issues of poor treatment engagement [36]. Pooled findings from prior research estimate that treatment dropout is 74% for unsupervised digital therapeutics [37], which is problematic as low engagement has been shown to reduce the efficacy [38]. The relatively high rates of treatment “completion” reported in the LifeBuoy condition (71.5%) does appear to support acceptability and relevance may be enhanced by codesign. Second, unlike this trial, neither of the previously referenced trials of smartphone applications [16,17] provided an application that exactly replicated the aesthetics and design of the interventional application to the control condition. Although Franklin and colleagues [16] did also use a control application, there is no evidence to suggest it mirrored the intervention program. By creating a robust digital placebo, we were able to test whether the therapeutic content contributed to improvements in key outcomes above and beyond participant expectations about LifeBuoy (i.e., the digital placebo effect) [39]. The potential for the digital placebo effect to influence efficacy is demonstrated in a prior study of a smartphone application for adolescents to self-monitor symptoms of depression; the study found that without any direct therapeutic intervention, self-monitoring significantly reduced symptoms [40]. As such, our trial design increases confidence the superior effects reported in our LifeBuoy condition are, at least in part, directly attributable to the treatment content itself. Not only does this study strengthen the evidence base for the short-term efficacy of brief unsupervised and targeted digital therapeutics in young adults [14], but it also demonstrates that LifeBuoy application has a favorable risk–benefit profile. Relative to Lifebuoy, the control condition had a 22% increase in the odds of flagging as high risk of suicidal behavior at postintervention. Also, no suicide attempts or serious adverse events were reported in the intervention condition at the final survey, signaling that LifeBuoy could be made available for young adult suicidal ideators without substantial safety concerns. To further understand the potential harms and benefits of LifeBuoy, however, this trial would have benefitted from including individuals with recent suicidal behavior. As a prior suicide attempt confers a significantly higher odds of experiencing a future suicide attempt compared to ideation [5], the inclusion of actively suicidal persons may have better positioned this study to detect serious adverse events (harms) and improve our understanding of the application’s potential to interrupt ideation-to-action processes and prevent future attempts [41]. Also, the inclusion of novel methods, such as ecological momentary assessment (EMA) and data linkage [42,43], in this trial would have improved the capture of real-time and long-term impacts of LifeBuoy on suicide ideation, attempt, and death, which would further strengthen causal inferences regarding the contribution of digital therapeutics to suicide prevention.

Close to 90% of participants had received treatment from a mental health professional prior to joining the trial. Rather than “reach the unreachable” (i.e., the nonhelp seekers [12]), this trial appeared to appeal to young adults with high rates of treatment familiarity and engagement. This finding is not new but has not received much attention; similar trials also report high rates of prior treatment engagement (60% or higher; [31,44]). This bias toward treatment engagers may be because young people are unwilling to disclose suicidal ideation mental health professionals. Prior research suggests that nondisclosure is not uncommon—and may be related to fear of stigmatization, uncertainty of repercussions, and prioritization of other mental health issues [45]. Accordingly, unsupervised digital interventions might provide a solution to these concerns, enabling young people to manage suicidal ideation in private. It will be important to explore the reasons why young people who are accessing mental health services would be motivated to engage with a targeted digital therapeutic for suicidal ideation. Such insights would help to inform decisions regarding best fit delivery pathways for enabling reach and access of digital therapeutics; if young adults are unwilling to disclose ideation to clinicians, then delivering these interventions through health systems and services may not be appropriate.

The high rates of prior treatment exposure in this study raise the question of whether treatment familiarity contributed to the effects of LifeBuoy on ideation, and whether it would work as well for those without such exposure. Certainly, the brief nature of smartphone interventions, necessitated by the physical constraints of screen sizes, creates potential for the meaning and actioning of complex therapeutic concepts to be lost in translation. Comparisons of efficacy trajectories of those with and without treatment exposure could provide novel insights into who these interventions work best for.

Several other limitations of the current study should be considered. Our sample was biased toward females, Lesbian, Gay, Bisexual, Transgender, Queer, and Intersex (LGBTQI) persons, and those who had previously received treatment. The targeted recruitment of males and nontreatment recipients using purpose designed Facebook advertisements could help address questions of generalizability. Only 1.7% of those who saw the Facebook advertisements initiated the screening for eligibility process, indicating a high rate of reach but low click conversion rates. Thoughtfully designed advertisements for specific populations may help improve conversion rates by enhancing appeal, while reconsideration of the targeted parameters of the advertisements (e.g., interests, demographics, and geography) may reduce the likelihood that they are seen by those who would be ineligible on key inclusion criteria [46].

Power analyses were calculated for our primary outcome, but not for any of our secondary outcomes or post hoc analyses. By the final survey, the attrition from assessment rate was 45%, consistent with prior clinical trials of smartphone interventions [36]. Attrition was associated with more severe baseline clinical profiles than those who remained in the study at T2. Substantial psychological effort is required to attain treatment goals—particularly for unsupervised treatment—and severe mental illness can be a major barrier to being able to sustain efforts. Individuals who dropped out may have benefitted less from our intervention, and without their actual data, efficacy may be overestimated. Strategies to improve retention should be explored in future trials. Given these limitations, the transfer of benefit of the LifeBuoy intervention to real-world settings and the full clinical meaningfulness of the findings, as well as the mechanisms underlying these effects, should be explored in further studies.

In conclusion, our findings indicate that a brief, unsupervised DBT smartphone intervention can reduce suicidal ideation in young adults, but these effects do not extend to improvements in other mental health issues. To confirm whether LifeBuoy could help to increase access to care for suicidal ideation among youth unable or unwilling to access face-to-face treatment or supplement other forms of mental health treatment, the results of this first trial need be replicated and extended to suicidal behavior in future clinical trials.

## Supporting information

S1 CONSORT Checklist CONSORT 2010 checklist of information. CONSORT, Consolidated Standards of Reporting Trials.
(DOC)

S1 Data LifeBuoy trial data. (XLSX)

S1 Fig Overview of the LifeBuoy smartphone application. (TIF)

S1 Protocol Clinical trial protocol for a randomized controlled trial of a mHealth intervention to help young people manage suicidal thoughts. (DOCX)

S1 Table Baseline characteristics of the sample for those who did or did not complete the T1 and T2 surveys. T1, postintervention; T2, 3-month postintervention.
(DOCX)

S1 Text Exploratory analysis of clinically significant change on suicidal ideation. (DOCX)

## Abbreviations:

ACCEPTS: Activities, Contributing, Comparisons, Emotions, Push away, Thoughts, and Sensation
CBT: cognitive behavioral therapy
CONSORT: Consolidated Standards of Reporting Trials
DBT: dialectical behavior therapy
DQ5: Distress Questionnaire-5
EMA: ecological momentary assessment
GAD-7: Generalized Anxiety Disorder-7
LGBTQI: Lesbian, Gay, Bisexual, Transgender, Queer, and Intersex
NNT: number needed to treat
OR: odds ratio
PHQ-9: Patient Health Questionnaire-9
SIDAS: Suicidal Ideation Attributes Scale
SWEMWBS: Short Warwick–Edinburgh Mental Well-Being Scale
TIPP: Temperature, Intense exercise, Paced breathing, Paired muscle relaxation
