# Supplementary figures and images for: The effect of a therapeutic smartphone application on suicidal ideation in young adults: Findings from a randomized controlled trial in Australia
Source: PLoS Med. 2022 May 31;19(5):e1003978. doi: 10.1371/journal.pmed.1003978 (PMC9154190; doi:10.1371/journal.pmed.1003978)

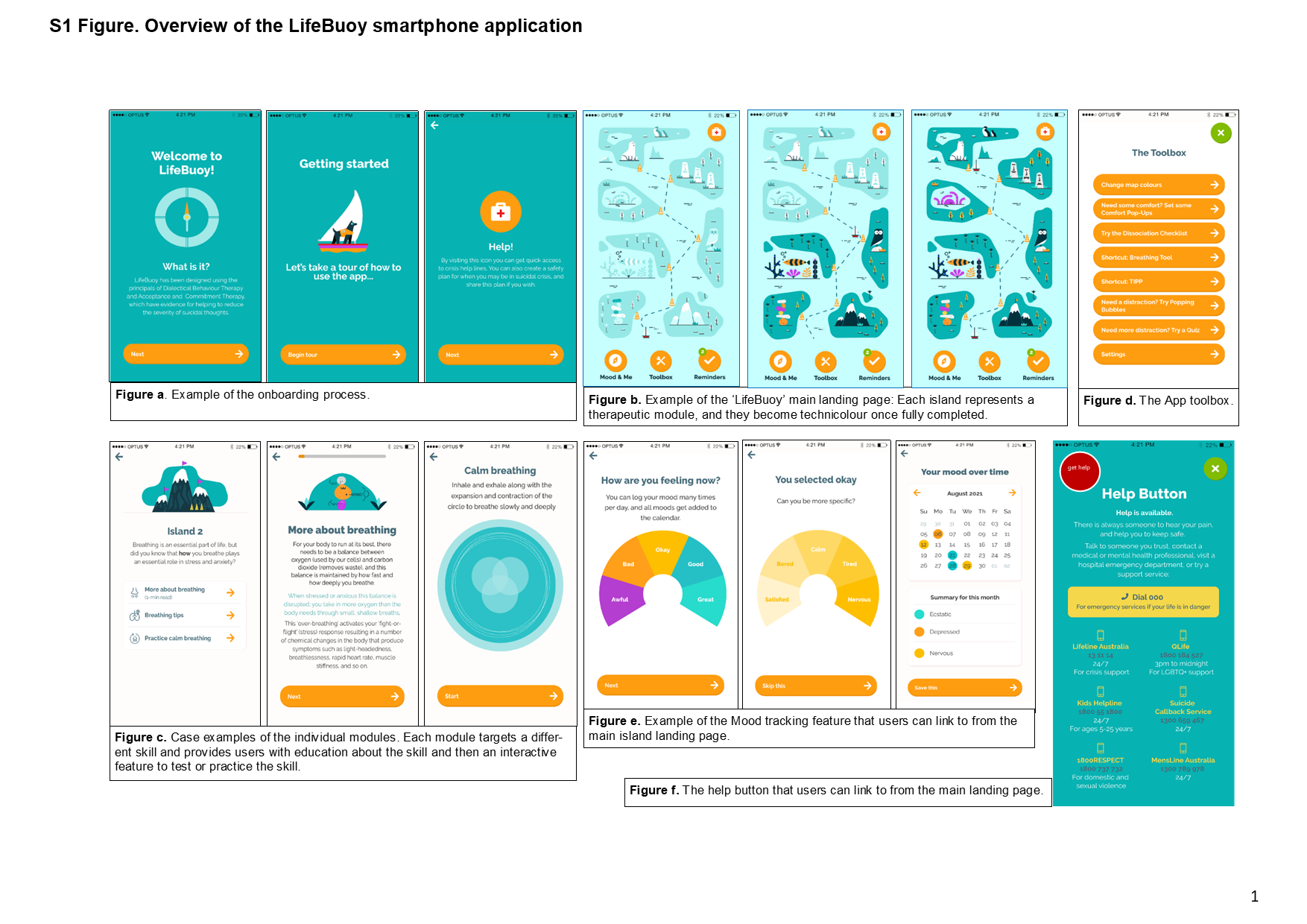

Supplement: S1 Fig — (TIF) [file pmed.1003978.s003.tif]
