# Supplementary material for: The effect of a therapeutic smartphone application on suicidal ideation in young adults: Findings from a randomized controlled trial in Australia
Source: PLoS Med. 2022 May 31;19(5):e1003978. doi: 10.1371/journal.pmed.1003978 (PMC9154190; doi:10.1371/journal.pmed.1003978)
Supplement: S1 Protocol — (DOCX) [file pmed.1003978.s004.docx]

**S1 Protocol**

**Physiological, Psychological, Psychiatric, Surgical or Health Interventions**

Clinical trial protocol for a randomised controlled trial of a mHealth intervention to help young people manage suicidal thoughts

Application number: HC190764

Dr Michelle Tye, UNSW Coordinating Principal Investigator

Principal Investigators: Jin Han, Quincy Wong (also study statistician), Aliza Werner Seidler, Bridianne O’Dea, Alison Calear, Helen Christensen.

Trial manager: Dr Lauren McGillivray

Trial registration number ACTRN12619001671156

The intervention has been registered with the therapeutic goods administration through the Clinical Trial Notification (CTN) scheme (CT-2020-CTN-00256-1-v1).

**Statement of Compliance**

The trial will be conducted in accordance with the National Statement on Ethical Conduct in Human Research (2007), the CPMP/ICH Note for Guidance on Good Clinical Practice and consistent with the principles that have their origin in the Declaration of Helsinki.

Compliance with these standards provides assurance that the rights, safety and well-being of trial participants are respected. This protocol was developed in accordance with the SPIRIT (Standard Protocol Items: Recommendations for Interventional Trials) 2013 Statement.

**Summary of Approvals and Modifications** (see Summary of Changes from Version 1.0)

Original Approved Version 1.0: 20^th^ January 2020

Modification Approval Version 1.1: 14^th^ March 2020

Modification Approval Version 1.2: 20^th^ April 2020

**Table of Contents**

[1. Study Objectives and Hypotheses 4](#_Toc95296271)

[1.1. Primary Aim 4](#_Toc95296272)

[1.2. Secondary Aim 4](#_Toc95296273)

[1.3. Tertiary Aims 4](#_Toc95296274)

[1.4. Hypotheses 4](#_Toc95296275)

[2. Study Background 5](#_Toc95296276)

[2.1. Rationale 5](#_Toc95296277)

[2.2. The LifeBuoy Study. 6](#_Toc95296278)

[3. Study Design 6](#_Toc95296279)

[3.1. Treatment Design 6](#_Toc95296280)

[3.2. Randomisation 7](#_Toc95296281)

[3.3. Study Population 7](#_Toc95296282)

[4. Selection and Enrollment of Subjects 8](#_Toc95296283)

[4.1. Inclusion and Exclusion Criteria 8](#_Toc95296284)

[4.2. Study Recruitment Procedures 8](#_Toc95296285)

[4.3. Consent and Screening 9](#_Toc95296286)

[4.4. Condition Allocation 10](#_Toc95296287)

[4.5. Withdrawal of Consent or Participant 10](#_Toc95296288)

[5. Study interventions 11](#_Toc95296289)

[5.1. LifeBuoy (intervention) 11](#_Toc95296290)

[5.2. LifeBuoy-C (control condition) 12](#_Toc95296291)

[6. Outcomes and Schedule of Surveys 12](#_Toc95296292)

[6.1. Primary outcome measure 12](#_Toc95296293)

[6.2. Secondary outcome measures 13](#_Toc95296294)

[6.3. Tertiary outcome measures 14](#_Toc95296295)

[6.4. Risk factors and other measures 15](#_Toc95296296)

[6.5. Assessment schedule 17](#_Toc95296297)

[7. Statistical Analysis 18](#_Toc95296298)

[8. Sample size 18](#_Toc95296299)

[9. Data Handling and Ownership 19](#_Toc95296300)

[9.1. Handling and Reporting Data 19](#_Toc95296301)

[9.2. Direct Access to Source Data and Documents 19](#_Toc95296302)

[9.3. Monitoring Quality Control and Quality Assurance 19](#_Toc95296303)

[10. Safety and Monitoring 20](#_Toc95296304)

[10.1. Assessment of Adverse and Safety Events 20](#_Toc95296305)

[10.3. Serious Adverse Events 21](#_Toc95296306)

[10.4. Significant Safety Issue (SSI) 21](#_Toc95296307)

[11. Non-compliance, Protocol Deviation and Serious Breaches of Good Clinical Practice 22](#_Toc95296308)

[11.1. Protocol Deviation 22](#_Toc95296309)

[11.2 Serious Breach of Good Clinical Practice 23](#_Toc95296310)

[11.3. Reporting Protocol Deviations 23](#_Toc95296311)

[11.4. Reporting of a Serious Breach 24](#_Toc95296312)

[11.5. Review of a Protocol Deviation and a Serious Breach 24](#_Toc95296313)

[12. Risk management 25](#_Toc95296314)

[12.1. Risk mitigation strategies 25](#_Toc95296315)

[12.2. Privacy, confidentiality, and data management 25](#_Toc95296316)

[13. References 26](#_Toc95296317)

[Appendix A: Recruitment materials 30](#_Toc95296318)

[Appendix B: Screening and Eligibility 32](#_Toc95296319)

[Appendix C: Survey Measures 34](#_Toc95296320)

[Appendix D: UNSW Safety Monitor Register Template 51](#_Toc95296321)

[Appendix E: Data and Safety Monitoring Board Charter 52](#_Toc95296322)

[Data and Safety Monitoring Board (DSMB) Overview 52](#_Toc95296323)

[Summary of Changes from Version 1.0 60](#_Toc95296324)

# Study Objectives and Hypotheses

## 1.1. Primary Aim

To assess the efficacy of the LifeBuoy app in reducing suicidal ideation in young people, as measured by the Suicidal Ideation Attributes Scale. Changes in suicidal ideation will be assessed by comparing the intervention and attention-matched (placebo) control conditions at baseline and post-intervention, and 3-month post intervention follow-up.

## 1.2. Secondary Aim

To assess whether the LifeBuoy app significantly reduces depression, anxiety, and psychological distress, and increases general mental wellbeing at post-intervention and 3-month follow-up, relative to the attention-matched (placebo) control condition.

## 1.3. Tertiary Aims

To examine the engagement with the smartphone application in each condition, assessed as uptake (no. downloads), adherence (no. modules completed) and acceptability among intervention condition participants.

To examine whether there is a dose-response relationship between engagement with the application and the primary outcome (suicidal ideation).

To assess whether the LifeBuoy app significantly reduces insomnia, rumination, suicide cognitions, distress tolerance, and help-seeking intentions and behaviours at post-intervention and three-month follow-up relative to control condition.

To examine whether loneliness, demographics, perseverance, negative events (i.e., such as COVID-19), and expectation of treatment success mediate the relationship between allocated condition and the primary outcome (suicidal ideation).

## 1.4. Hypotheses

H1: Participants in the intervention condition will report greater reductions in suicidal ideation scores at post-intervention compared to the placebo control condition.

H2: Participants in the intervention condition will report greater reductions in depression and anxiety symptoms at post-intervention than the control condition.

H3: Higher levels of adherence to the LifeBuoy app will be associated with greater reductions in suicidal ideation at post-intervention.

# Study Background

## 2.1. Rationale

Youth suicidal ideation and behaviour remain a major public health concern because of their life-threatening nature and widespread prevalence. It is an issue that exists across psychiatric diagnoses and in varied socio-cultural populations. In Australia, suicide is the leading cause of death among those aged 15 to 44 years [1]. The importance of the problem of suicidality is further highlighted by its prevalence in the general population. Suicidal ideation is relatively common among adolescents, with 12-month prevalence estimates as high as 24% in those aged 18 or younger [2]. Over one‐third of adolescents who experience suicidal ideation go on to attempt suicide [3]. Youth suicide also carries significant economic loss, which is estimated to be $511M per annum [4].

The extent of suicidality is, at least in part, compounded by adolescents’ reluctance to seek help for psychological distress and suicidality. A recent review [5] reported that of 12,006 individuals with past-year suicide ideation, plans, and/or attempts, the weighted average prevalence of seeking or engaging with mental health services was 29.5%. Lower rates of help- seeking were associated with being younger, being male, and cultural influences [5,6]. Potentially up to 70% of young people have not sought or received access to mental health services. Traditional face-to-face treatment services also have numerous economic, logistical, and personal barriers (i.e., stigma) that prevent most young Australians from accessing them [7]. Digitally delivered interventions offer a new opportunity to detect at risk young people, improve access to support, and provide high fidelity/cost-effective treatment, addressing gaps observed in current population and health system approaches.

There is emerging evidence that adults readily access, and benefit from, suicide prevention interventions delivered via smartphone-based apps [8]. However, there have been very few empirical studies examining the efficacy of mental health apps for young people, and there are even fewer studies for apps that specifically target suicidal thoughts in this population. Of those interventions which have undergone RCT testing, only two have been mHealth (mobile phone, tablet) programs (and none specifically for young people), despite estimates that 94% of young people own a smartphone and that they spend up to one-third of their day using electronic devices (phones, computers) [9]. Considering these levels of smartphone ownership and use it is surprising that they remain relatively unexplored as a digital health solution in suicide prevention. To address this gap, we developed the LifeBuoy app, a self-help smartphone app designed to help young adults manage suicidal thoughts and negative feelings in daily life. It includes seven structured therapeutic sessions derived primarily from Dialectical behaviour therapy (DBT), which has been shown to reduce suicide-related outcomes, including non-suicidal self-injurious behaviour (NSSI) and suicide attempts [10,11]. DBT combines principles from behaviorism, Zen, and dialectics [12] that aim to help clients improve their emotional and cognitive regulation to overcome problems, including intense mood change [13,14], impulsivity [15,16], and loneliness [17]. DBT was initially developed for persons diagnosed with borderline personality disorder [18], and there is increasing evidence to suggest that DBT is also effective in reducing suicidal thoughts, NSSI, and suicide attempts in both adults [19] and adolescents [20,21]. To our best knowledge, no studies have investigated the possibility of a using a DBT-informed app to reduce suicidal thoughts in young adults.

We also recognise that the use of mental health digital interventions is highly variable between 21% - 88% engaging in minimal use, and 7% – 42% engaging in moderate use [22]. To overcome challenges with engagement, young people with a lived experience of suicide were surveyed and interviewed to identify features, content, and aesthetics that appealed to the target age group (e.g. available offline, well-organised and logically-linked content, youthful language, bright colours, and, positive illustrations, pace personalisation, self-monitoring and progress feedback) as factors that would improve engagement and adherence. These principles have been embedded in the LifeBuoy app, to minimise the risk of trialling an app that will not be used, or which is used very minimally.

## 2.2. The LifeBuoy Study.

The LifeBuoy study aims to evaluate whether a therapeutic smartphone application is effective and acceptable for reducing suicidal ideation l in young adults, aged 16 to 25 years who have experienced suicidal ideation in the past 12 months and. It tests an interventional smartphone application (LifeBuoy) against a matched attention control application (LifeBuoy-C) so that individuals will not know which app they have downloaded. The findings of the current study are important in providing initial evidence of whether smartphone applications are able to deliver accessible health interventions to young people.

# Study Design

## 3.1. Treatment Design

The flowchart (Figure 1) summarizes this study which will consist of evaluating the efficacy of a digital therapeutic for suicidal ideation, using a is a two-arm, parallel, double-blind superiority randomised controlled trial design. Following completion of the baseline (T0) assessment, participants will be randomised at equal allocation (2:2) to one of two smartphone applications, a DBT-based application (LifeBuoy) or a placebo (non-therapeutic) control application (LifeBuoy-C). After completion of the 6 week intervention, primary, secondary and tertiary outcomes will be assessed at immediate post-intervention (T1) and then at 4-months post-intervention (T2). All surveys will be completed online, with survey links distributed automatically to registered participants using Black Dog Institute’s research engine. Subjects in the intervention condition will be invited to participate in a qualitative intervention at the final assessment to understand their experience with the LifeBuoy application.


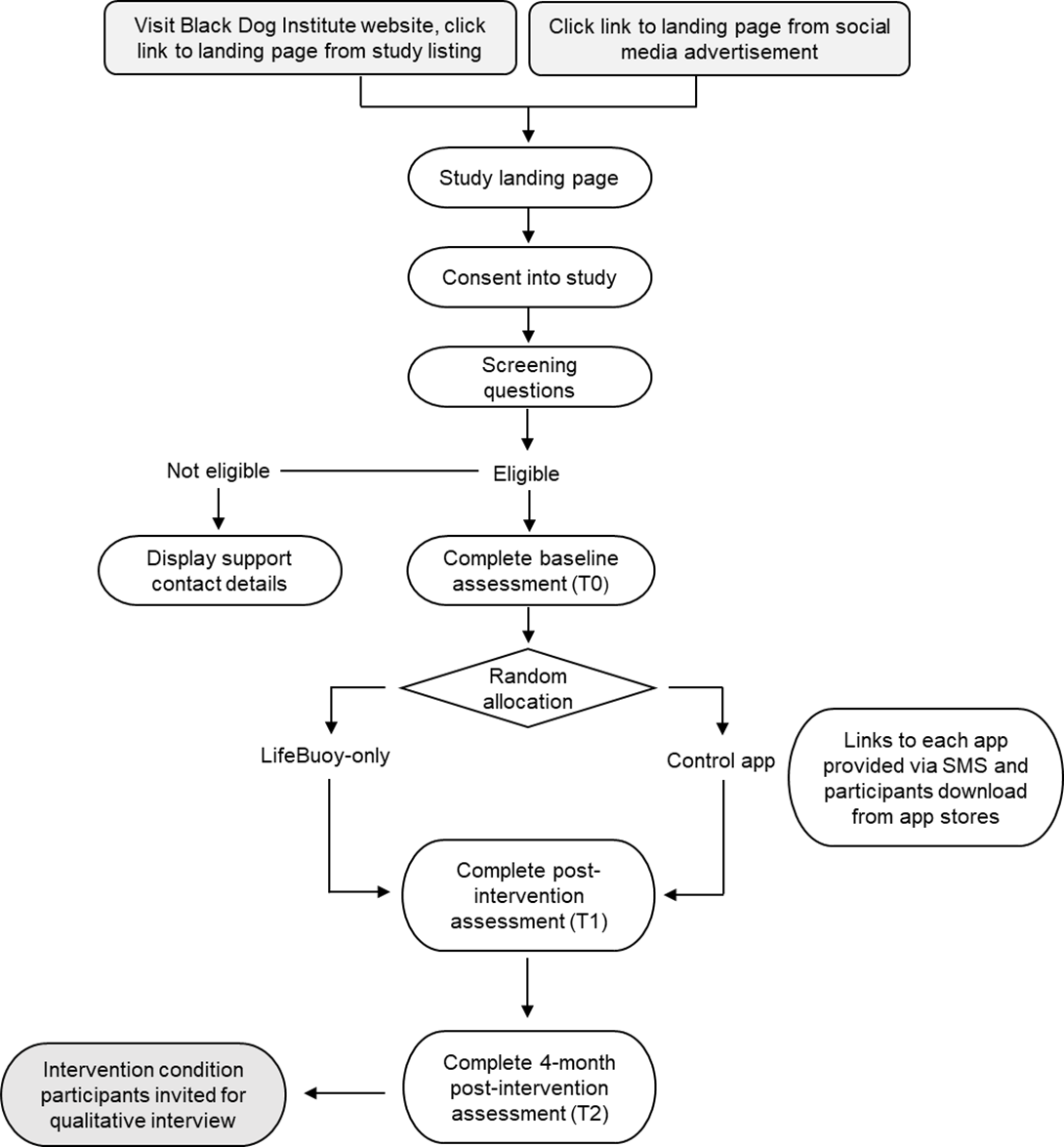


**Figure 1. Study flow**

## 3.2. Randomisation

Randomisation to the active or control condition will occur on a 2:2 ratio using a block design (4 participants per block) using an automated web-based platform tailored for this project. Randomisation codes will be generated and retained automatically by the Black Dog Institute’s bespoke trial software, the research engine.

## 3.3. Study Population

Our sample will consist of 378 community-based young adults aged between 16 and 25 years who have experienced suicidal ideation in the past year, recruited using an online, social media strategy. The overall goal of the inclusion/exclusion criteria is to identify a non-clinical population of suicidal ideators.

# Selection and Enrollment of Subjects

## 4.1. Inclusion and Exclusion Criteria

The inclusion criteria are as follows and will be determined by an online self-reported screening survey.

Participants will be deemed **eligible** if they:

- Are 16 to 25 years old (inclusive)
- Are living in Australia
- Are fluent in English
- Own and have access to a smartphone
- Experienced mild to moderate suicidal thoughts in the past year (SIDAS scores < 21).

Participants will be **ineligible** if they:

- Have ever been diagnosed with psychosis or bipolar disorder
- Have made a suicide attempt in past month.

## 4.2. Study Recruitment Procedures

**Trial.** Participants will be recruited into this trial via social media advertisements (e.g. Facebook, Twitter, Instagram, website), which are targeted to the inclusion criteria. Individuals interested in participating will click on the following link, which will be embedded in the advertisements:

https://blackdoginstitute.org.au/lifebuoy_study.

This link will take participants through to the study website hosted on Black Dog Institute’s research engine. Here they will be presented with an online version of the participant information sheet and consent form. Participants will need to consent to participation before being directed on to the eligibility screening survey. This strategy has been successfully used in a recent trial of a relationship app for depression, and 399 eligible participants were recruited and registered in one month. 71 (17.7%) of those recruited completed baseline assessments, indicating that the required sample size for this trial will take six months.

Recruitment wording in described in Appendix A.

**Qualitative Interviews.** For the qualitative interviews conducted at the final (T2) assessment, we will recruit a 10% subsample of individuals who participated in the intervention condition (expected to recruit n=189) to assess their experience with the app. Justification for this sample size is described in Section 8.

Recruitment to the qualitive interviews will take place immediately *before* the final assessment. At the time of the final assessment, but prior to receiving the assessment, participants in the intervention group will be emailed a letter of invitation to participate in the qualitative study, using the details they registered for the trial with. The delivery of this invite will be automated as part of a pre-determined schedule of survey and reminder delivery in the Research Engine.

The wording for the letter of invitation is as follows:

**“Before you start the final assessment, we just want to check if would you be interested in participating in a 30-minute one-on-one telephone or videoconference interview about your experience of using the LifeBuoy app? We are keen to hear from you if you did or did not use the app, to learn why. You will be reimbursed $30 for your time if you chose to participate:**

**Yes**

**No**

If a participant selects 'yes' a message will be displayed saying “**That is great news, we’ll now ask you to read and reply to the Participant Information and Consent Form, and then you can proceed on to the final assessment**”. Once they have read the Participant Information Sheet and completed the online consent, an automated email is sent to the research inbox (pts@blackdog.org.au) to notify the research team which participants to schedule an interview with.

If they select ‘no’, a message will be displayed saying “**Thanks for that, you can now proceed to the final assessment”** and will be able to click through to the survey.

As part of the Information Sheet, participants will be informed that they will be selected to participate in an interview on a first come first served basis, in case a large number of people are interested. Participants will be contacted by a research team member, and a date and time will be arranged for a telephone or videoconference interview (whichever they prefer). Participants will be told that if they fail to attend the interview at the designated time without giving prior notice, their place will be given to someone else.

Participants who respond after the first 20 people are confirmed will be informed by email that the quota is full, but they will be placed on a waiting list should some participants not be able to attend the interview. The order of the waiting list is ranked according to the participants’ response date and time, with earlier responses ranked higher on the list.

## 4.3. Consent and Screening

Participants will be provided with the Participant Information Sheet and Consent Form [PISCF] online, once they click on the recruitment advertisement. Participants will be asked to read the PISCF and will have sufficient time to consider their participation because there is no time restriction before they consent and register to participate. Participants will be advised to contact the researcher(s) if they have any questions. The PISCF will be available for participants to download at the time of reading and from the study landing page if they wish. Once they are comfortable providing their consent to participate, participants will be asked to digitally sign the consent form and click ‘submit’, which will all be managed online via the trial portal (hosted on Black Dog Institute’s research engine).

After the completing the consent procedure, potential participants will be automatically directed to complete a brief online survey within the study portal (website) to assess their eligibility for the trial. Individuals will be asked questions that align to the inclusion and exclusion criteria, including their age, whether they are living in Australia, fluency of English, smartphone ownership, previous diagnosis of psychosis or bipolar disorder, presence of suicidal thoughts and attempts. Individuals who are deemed ineligible will be directed to a webpage thanking them for their time. This webpage will include relevant support contact details. The screening questions and eligibility/ineligibility wording is described in Appendix B.

Young people who are eligible will be automatedly directed to register in the study (by providing an email address and mobile phone number), which will allow survey links and reimbursement to be digitally sent to them.

## 4.4. Condition Allocation

After screening and registration is complete, participants will be asked to complete the baseline assessment – which is also delivered online, via the trial portal (website). Upon completion of the baseline survey, they will be automatedly randomised to a condition via an algorithm built into the Research Engine trial software. Subjects are not informed of which condition they have been allocated to. The two applications both have LifeBuoy in their title and look the same, only the content differs.

## 4.5. Withdrawal of Consent or Participant

Participants may be withdrawn from the study if:

- They experience a serious adverse event that:
  - is attributable to a study intervention or procedure; or
  - means they can no longer participate in the study; or
- They withdraw consent to take part.
- We will not be replacing participants that are withdrawn from the study.

The team clinical psychologist will follow-up with subjects withdrawn due to experiencing a serious adverse event that was attributable to the study intervention or procedure. These participants will be contacted via email within 72 hours of withdrawing from the trial with a list of crisis contact numbers for support. In this email, participants will also be asked to respond to the email (by clicking ‘reply’) if they would like to receive a phone call from the team clinical psychologist during business hours (9:00 to 17:00 AEST). If they say yes, they will be contacted within the following 72 hours.

Participants can self-withdraw at any time by submitting to the study team the UNSW standard withdrawal form appended to the Participant Information Statement and Consent Form. No mechanisms for automated withdrawal (e.g. via the study app) will be provided. To ensure that data belonging to a participant who withdraws can be uniquely identified, their mobile number and email address will be solicited on the withdrawal of participation form. Excluding self-withdrawals, the decision to withdraw a participant will be made by the Principal Investigator.

Handling of participant data after withdrawal will depend on when a participant decided to withdraw from the study. Participants will have their data removed from the study if they:

- Withdraw or are withdrawn prior to completion of the baseline assessment. Reflecting the intention-to-treat principles informing the planned analyses of the trial, data from participants who withdraw after this point will not be discarded.

For those who install the app, access the app once, and complete baseline, subsequent non-response (rather than active withdrawal) will not be treated as withdrawal but handled as missing data unless the specifically request to have their data withdrawn.

Non-identifiable data about withdrawals enabling aggregate reporting (e.g. counts by intervention assignment) will be retained in a Withdrawals Log. In addition, any participant withdrawal linked to an adverse event will be recorded to enable reporting of safety-related statistics. Participants who withdraw from the study will not be replaced and will not be followed up except per adverse event follow-up, if applicable.

# Study interventions

## 5.1. LifeBuoy (intervention)

The Lifebuoy app is a smartphone application developed by researchers at the Black Dog Institute. It is a fully automated, self-help program for young people experiencing mild-to- moderate symptoms of suicidal ideation. The app has been developed using a person-centered approach, to understand and accommodate the perspectives of young people who will use the intervention.

Participants will download the app from the App Store or Google Play onto their personal smartphones. Once the app is downloaded, it will not require internet connection; internet connectivity will only be required to upload usage and adherence data to UNSW servers.

The app contains seven learning modules derived from Dialectical Behaviour Therapy (DBT) and incorporates behavioural activation principles (distress tolerance, emotion regulation/mindfulness, interpersonal effectiveness) to help young people develop strategies and problem-solving skills for managing suicide thoughts and distress.

Participants will have 6 weeks to complete the seven modules, each module will take approximately 3 to 7 minutes. The user will be directed to each module linearly, and, unlocking a new module will require completion of the previous one (this also is the gamification of the app, as island ‘light up’ or become technicolour when complete).

## 5.2. LifeBuoy-C (control condition)

For our control condition we created a sham app, LifeBuoy-C, which is designed to match LifeBuoy on expectancy and time on task to control for digital placebo effects. Similar to the intervention app, the control app contains seven brief non-therapeutic education-based modules. The topics are peripherally related to mental health and wellbeing, including confidence, performance stress, the importance of having goals, and the value of being present. Each module takes two to three minutes to read through. The control does not contain the toolbox but includes the help button for safety management. Participants in the control group will be granted access to the intervention app after they complete the three-month follow-up assessment.

At the completion of the final assessment, participants in the control condition will receive an email letting them know they received the non-therapeutic app (Appendix G). The email explains what being in the control condition meant, and why it is important to have a control condition when trying to establish the efficacy of an intervention. Within this email, they will be offered the opportunity to download, and have access to, the Lifebuoy therapeutic app for 6 weeks. The link to the app download will be embedded in the email. This will be the only communication regarding the app download, no additional reminders will be sent. Participants will be able to download the app at any point during that 6 week period. If they attempt to download it after the 6 weeks they will receive the following email: “Oops, looks like the link to the LifeBuoy has expired as it’s been longer than 6 weeks. We’re really sorry about that. If you’re not feeling great, please speak to your GP or call a crisis help line, such as LifeLine 13 11 14 or the Suicide Call Back Service 1300 659 467”.

# Outcomes and Schedule of Surveys

## 6.1. Primary outcome measure

**Suicidal Ideation Attributes Scale**

The primary outcome measure is the severity of suicidal thoughts assessed by the Suicidal Ideation Attributes Scale (SIDAS; van Spijker, Batterham [23]). It consists of five questions pertaining to frequency of suicidal thoughts in the past month, controllability of suicidal thoughts, closeness to suicide attempt, level of distress associated with the thoughts, and impact on daily functioning. Each item is assessed on a 11-point scale (0-10). Item two (controllability) is reverse scored. Total scale scores on the SIDAS range from 0 to 50, with higher scores indicating more severe suicidal thoughts.

## 6.2. Secondary outcome measures

**Suicide Behaviours**

Participants’ previous suicide attempts and self-injury are assessed by eight questions developed for a previous suicide prevention trial [23]. Participants are invited to indicate whether they have attempted suicide in their lifetime and in the past 30 days on a three-point Likert scale, ranging from ‘No, never (0)’, ‘Yes, once (1)’, to ‘Yes, more than once (2)’. They are also asked to report the number of suicide attempts over their lifetime, and the number of months that they have been thinking about suicide. Apart from that, participants are asked to indicate whether they have intentional self-injury in their lifetime on the aforementioned three-point Likert scale. If yes, they are asked to provide the number of times of their intentional self-injury and rate the severity of the worst injury in the past month on a three-point Likert scale, ranging from ‘No care was needed (1)’, ‘Some care was needed (2)’, to ‘Required medical care (3)’.

**Depression symptoms**

Patient Health Questionnaire-9 (PHQ-9; Kroenke, Spitzer [24]) is a nine-item self-report questionnaire measuring the severity of depression. The scale assesses the frequency of occurrence of depression symptom in the previous two weeks, with items rated on a four-point scale ranging from ‘Not at all (0)’ to ‘Nearly every day (3)’. Total scores on the PHQ-9 depression scale can range from 0 to 27, with higher scores reflecting more severe depression.

**Generalised Anxiety Symptoms**

The Generalised Anxiety Disorder-7 (GAD-7; Spitzer, Kroenke [25]) is a seven-item self-report measure designed to assess the severity of generalised anxiety symptoms over the previous two-week period. Items are responded to a four-point scale, ranging from ‘Not at all sure (0)’ to ‘Nearly every day (3)’. Total scores on the GAD-7 can range from 0 to 21. Higher scores indicate higher levels of GAD symptoms.

**Psychological Distress**

The Distress Questionnaire-5 (DQ-5; Batterham, Sunderland [26]) is a five-item brief screening tool for identifying general psychological distress. Participants are asked to endorse the frequency of each item in the past 30 days on a five-point scale ranging from ‘Never (1)’ to ‘Always (5)’. Total scores range from 5 to 25, with higher scores indicating greater psychological distress.

Short Warwick-Edinburgh Mental Wellbeing Scale

**Wellbeing**

The Short Warwick–Edinburgh Mental Well-Being Scale (SWEMWBS; Fat, Scholes [27,28] is a shortened seven-item version of the 14-item Warwick–Edinburgh Mental Well-Being Scale (WEMWBS) [28], which was developed to assess mental well-being in the general population. It assesses mental wellbeing by asking participants’ feeling and experience over the previous two weeks. Responses range from ‘None of the time (1)’ to ‘All of the time (5)’ and raw item-scores are summed and converted to a metric total score using the SWEMWBS conversion table [35]. Total scores can range from 7 to 35, with higher scores indicating higher levels of mental well-being.

## 6.3. Tertiary outcome measures

**Insomnia Symptoms**

The Insomnia Severity Index (ISI; Morin, Belleville [29]) is a psychometrically sound, seven-item self-report measure assessing the perceived severity of insomnia symptoms, the degree of satisfaction with sleep, interference with daytime functioning, noticeability of impairment, and concern caused by the sleep problems in the previous two weeks. Responses are reported on a five-point scale yielding total scores of 0 to 28. Higher scores indicate greater insomnia severity.

**Repetitive Thinking**

The Repetitive Thinking Questionnaire (RTQ-10; McEvoy, Mahoney [30]) is a transdiagnostic measure of engagement in repetitive negative thinking following distressing situations. The RTQ was developed to capture the underlying construct of recurrent negative thinking underlying mental health disorders such as depression and anxiety. Participants are requested to respond to the RTQ-10 on a five-point Likert scale ‘Not true at all (1)’ to ‘Very true (5)’. Total scores fall between 10 and 50, higher scores indicating greater rumination.

**Suicide Cognitions**

The shortened version of Suicide Cognitions Scale (SCS; Bryan, Kanzler [31]) is a self-report instrument consisting of nine items that are designed to measure suicide-specific cognition. The items contain statements consistent with the suicidal schemas of unbearability (e.g., ‘I can’t cope with my problems any longer’), unlovability (e.g., ‘I am completely unworthy of love’) and unsolvability (‘Nothing can help me solve my problems’). Items on the SCS are rated on a 5-point Likert scale from ‘Strongly disagree (1)’ to ‘Strongly agree (5)’. The instrument is scored by summing ratings across items, resulting in scores ranging from 9 to 45.

**Distress Tolerance**

The Distress Tolerance Scale (DTS; Simons and Gaher [32]) is a 15-item self-report measure designed to assess respondents’ perceived capacity to experience and endure negative emotional states. The DTS encompasses four subscales, including tolerance, appraisal, absorption, and regulation. Items are rated on a five-point Likert scale from ‘Strongly disagree (1)’ to ‘Strongly agree (5)’. Higher mean scores indicate a greater tendency to withstand emotional distress.

**Loneliness**

The Three-Item Loneliness Scale (TILS; Hughes, Waite [33]) is a brief self-report measure of loneliness. The three items that compose this scale were selected from the R-UCLA Loneliness Scale (Russell et al., 1980) and include: ‘How often do you feel that you lack companionship?’ (relational connectedness); ‘How often do you feel left out?’ (collective connectedness); and ‘How often do you feel isolated from others?’ (general isolation). Response categories for the TILS are: ‘Hardly ever (1)’, ‘Some of the time (2)’, and ‘Often (3)’. Total scores are calculated by summing item scores, with higher scores indicating greater loneliness.

**Help-Seeking**

The General Help-Seeking Questionnaire (GHSQ; Wilson, Deane [34]) is used in the current trial to assess participants’ intentions to seek help for suicidal thoughts from a variety of sources. Respondents are invited to rate, on a six-point scale from ‘Not applicable (0)’, ‘Extremely unlikely (1)’ to ‘Extremely likely (5)’, the likelihood of seeking help from three professional sources (school or university counsellor, mental health professional, Doctor/GP), four informal sources (boyfriend/girlfriend, friends, parents, other relative/family members), three telephone/online sources (phone helpline, internet website, mobile app) or no one. An optional item, ‘I would seek help from another source not listed above,’ is also provided. Higher scores represent stronger intentions to seek help.

The adapted version of Client Service Receipt Inventory (CSRI; Beecham and Knapp [35]) is designed to collect information about use of healthcare and social care services over a retrospective period of the past six months. Respondents are asked to indicate whether they have used any services in the past six months due to mental health problems, including suicidal thoughts on a binary scale (‘Yes’/’No’). Services include: hospital services, mental health helpline, crisis support team, police/ambulance, contact with a range of mental health professionals (e.g., social worker, counsellor), self-help group, and other medically qualified doctor.

## 6.4. Risk factors and other measures

**Demographic Information**

At the baseline assessment, participants are asked to provide their age, gender identity, gender assigned at birth, sexual orientation, contact information (email and mobile number), the state and area they live in (i.e., metropolitan or rural/remote), language spoken at home, who they live with at home, current relationship status, the highest level of education completed, employment status, and whether they have ever experienced or been diagnosed with mental illness. Information related to their service use is also collected, such as whether they have ever seen a mental health professional for a mental health problem and use of health or wellbeing apps.

**Grit**

The Short Grit Scale (SGS; [36,37]) is an eight-item measure assessing perseverance and passion for pursuing long-term goals. Half of the items are worded positively (e.g., ‘I am diligent’) while the other half are worded negatively (e.g., ‘New ideas and projects sometimes distract me from previous ones’) and are thus reverse scored. Items are rated on a five-point scale, ranging from ‘Not like me at all (1)’ to ‘Very much like me (5)’, with total scale scores ranging from 8 to 40.

**Negative Life Events**

The Negative Life Events Scale for Students (NLESS; Buri, Cromett [38]) is designed to assess the experience of stressful life events among students. Respondents are asked to indicate whether or not they have experienced 25 negative life events (e.g., death of a family member, being arrested) in the past year and if yes, how stressful that event has been for them. In the adapted version of this measure, items are rated on a five-point scale, ranging from ‘Not stressful (1)’ to ‘Extremely stressful (5)’. Higher mean scores indicate higher negative impact on life.

**Expectation of Treatment Success**

Four items were created to measure participants’ confidence and readiness in using an app to reduce suicidal thoughts, as well as the perceived importance of reducing suicidal thoughts and participating in research to reduce suicide risk. Items on this scale include ‘I am confident that people could reduce their suicidal thoughts using an app’ and ‘I think that participating in a study that aims to reduce suicidal thoughts is an important thing to do’. These items are rated on a five-point scale (ranging from ‘Strongly disagree (1)’ to ‘Strongly agree (5)’). Other items include ‘Please rate the importance of reducing your suicidal thoughts over the next six months’ (ratings range from ‘Not important (0)’ to ‘Very important (5)’) and ‘Please rate your readiness to reduce your suicidal thoughts by using an app’ (ratings range from ‘Not ready (1)’ to ‘Completely ready (5)’). Due to the nature of these questions, this measure is administered only once during baseline assessment. Total scores indicate higher expectation of treatment success.

**Satisfaction with the intervention**

Eighteen items were adapted from a previous study to assess participants’ satisfaction with the LifeBuoy app [39]. This measure consists of three parts. The first part contains seven statements related to the usability, readability, and helpfulness of the app, and the respondent’s intention to continue to use and recommend the app. Participants are asked to indicate whether they agree or disagree with each item. The second part of this measure comprises ten questions pertaining to potential difficulties in using the app (e.g., forgetting to use it, feeling worse after using it). Participants are asked to indicate whether they agree or disagree with each item. In the last part of this measure, they are asked to rate the overall helpfulness of the app on a five-point scale, ranging from “Extremely unhelpful (1)” to “Extremely helpful (5)”. Higher scores on each item indicating higher satisfaction with the app.

**Engagement/Treatment Adherence**

This will be measured by the number of modules accessed and completed by participants, and the time spent on each module. This data is automatically collected via the app.

## 6.5. Assessment schedule

Data will be collected at baseline (day 0), post-intervention (day 42), and 4-month follow-up (day 182).The Table below describes the timing of each measure. All survey questions/items are described in Appendix C.

**Table. Overview of Survey Schedule**

| Measure | **Outcome** | **Screening** | **Pre** | **Post** | **4-month** |
| --- | --- | --- | --- | --- | --- |
| Demographics | Demographics | - | X | - | - |
| SIDAS | Suicidal ideation | - | X | X | X |
| PHQ-9 | Depression, suicide attempt | - | X | X | X |
| GAD-7 | Anxiety | - | X | X | X |
| DQ-5 | Psychological Distress | - | X | X | X |
| SGS | Suicide Cognitions | - | X | X | X |
| RTQ-10 | Rumination | - | X | X | X |
| ISI | Insomnia | - | X | X | X |
| SWEMWBS | General mental wellbeing | - | X | X | X |
| DTS | Distress tolerance | - | X | X | X |
| TILS | Loneliness | - | X | X | X |
| GHSQ | Help-seeking intention | - | X | X | X |
| Modified-CSRI | Actual help- seeking | - | X | - | X |
| Modified-NLESS | Negative life events | - | X | - | - |
| SGS | Grit | - | X |  |  |
| Expectations of Success | Expectations of Success | - | X | - | - |
| Satisfaction with app | Satisfaction | - | - | X | - |

Participants in both conditions will be reimbursed for undertaking the post-intervention(T1) and 4-month post-intervention (T2) follow up surveys to reduce attrition at these timepoints. Participants will receive a $20 e-giftcards (from GiftPay; see <https://www.giftpay.com.au/business/egifts.aspx>.) at each of these two timepoints, providing a total reimbursement of $40 per participant if they undertake both surveys.

# Statistical Analysis

Mixed models repeated measures analyses, with maximum likelihood estimation and an appropriate covariance structure, will be used to evaluate the efficacy of the Lifebuoy app relative to the control condition. The primary outcome is severity of suicidal ideation as assessed by the Suicidal Ideation Attributes Scale [SIDAS] over time (pre- test to post-test; pre-test to follow-up). The mixed models approach incorporates all available data, including participants with missing follow-up data points, under the missing- at-random assumption. Analyses will therefore accord with the intention-to-treat principle. This same approach will be used to examine reductions in secondary outcomes (depression (PHQ-9) anxiety (GAD-7) psychological distress (DQ-5) and wellbeing (SWEMWBS)) and tertiary outcomes (rumination (RTQ-10) insomnia (ISI) general mental wellbeing (SWEMWBS) help-seeking (GHSQ, CSRI)) over time.

Potential mechanisms of the symptom reduction will be measured by the changes in the levels of distress tolerance (DTS), loneliness (TILS) or suicide cognition (SGS). Negative life events (NLESS), grit (SGS) and questions about expectation of treatment success are included in the surveys as potential moderators. Participants’ current medications/interventions will be controlled for data analysis.

Descriptive statistics will be used to evaluate app engagement for participants in both conditions. Adherence will be indicated by the number of modules completed by participants. Participant module access and module completion will be automatically collected via the app. The mixed model approach will be used to determine whether adherence moderates the change in SIDAS scores over time in the intervention condition.

Acceptability of the LifeBuoy app will be examined through semi-structured interviews with a subset of participants and questionnaires in the survey. The interview data will be analysed using thematic analysis [40]. An inductive approach, independent of a theoretical confirmative method will be used to identify and group themes. The researchers will then refine the themes and determine the final coding framework. Discrepancies will be resolved by a third researcher to ensure reliability of the process.

# Sample size

Based on a systematic review and meta-analysis [8], an effect size of 0.30 (Cohen’s d) will be expected between the intervention condition and the control condition at post-test in terms of suicidal ideation as measured by the SIDAS (Appendix C). Based on an attrition rate of 30% and a 0.50 correlation between pre- and post-test SIDAS scores, a sample size of n = 189 in each condition (total N = 378) will detect this expected effect size with power = 0.80 and alpha = 0.05. The planned sample size of n = 189 for the intervention condition will also allow statistical power of at least 0.80 to detect small-to-medium size effects in moderation analyses.

For the qualitative interviews we aim to recruit 10% of participants from the Intervention condition. This will result in a subsample of qualitative interviews of approximately n=20 persons based on a recruitment target of n=189 in each condition. In prior qualitative studies that have examined users’ experience with an app for managing psychological and physical health conditions, the sample sizes ranged from between 15 and 25 participants [41 – 43] which indicates our sample size is acceptable and justifiable.

# Data Handling and Ownership

All research data collected during this trial is governed and handled following the Research Data Governance and Materials Handling policy. UNSW, rather than any individual or Organisational Unit, is the Custodian of data and materials and any information derived from the data. Original research data and primary materials generated in the research conducted at the University will be owned and retained by the University subject to any contractual, statutory, ethical, or funding body requirements.

## 9.1. Handling and Reporting Data

Principal Investigators are responsible for maintaining adequate and accurate source documents and trial records that include all pertinent observations on each site's trial subjects. Source data must be attributable, legible, contemporaneous, original, accurate, and complete.

Trial subjects will be assigned a unique participant ID following registration. Data reported on the case report form, derived from source documents, should be consistent with the source documents, or the discrepancies must be explained. Any change or correction to a case report form should be dated, initialled, and explained (if necessary) and should not obscure the original entry (i.e., an audit trail should be maintained); this applies to both written and electronic changes or corrections.

## 9.2. Direct Access to Source Data and Documents

Site principal investigator(s) and institution(s) will permit trial-related monitoring, audits, IRB/IEC review, and regulatory inspection(s), providing direct access to source data/documents.

# 9.3. Monitoring Quality Control and Quality Assurance

The Coordinating Principal Investigator and Principal Investigator(s) 'responsibility are to monitor the clinical trial. The Coordinating Principal Investigator and Principal Investigator(s) are responsible for undertaking or participating in site initiation or protocol-specific training before recruitment and data collection commences. A monitoring report demonstrating regular compliance monitoring with the clinical trial protocol, procedures is provided to the UNSW HREC annually.

Root, cause, analysis reports are to be completed by the Coordinating Principal Investigator for reports of non-compliance and serious breaches. A corrective and preventative action plan must be developed and actioned for any reports of non-compliance and serious breaches.

# Safety and Monitoring

## 10.1. Assessment of Adverse and Safety Events

All adverse events and safety issues will be reported as per the Safety Monitoring Reporting Guidelines for Clinical Trials set out by the UNSW Human Research Ethics Committee. All adverse events and serious adverse events defined below will be reported by the Coordinating Principal Investigator to the Data Safety Monitoring Board (DSMB) and the UNSW Human Research Ethics Committee using the guidelines indicated in Table 2.

a. Known Adverse Effects

An individual scoring above 20 on the SIDAS will be considered an expected adverse event (given the study population has a history of recent suicidal ideation).

b. Known Harms, Risks or Discomforts

- Anxiety induced by answering a questionnaire and/or engaging in therapeutic activities on the app at the end of the data collection phase. There is a moderate likelihood and low severity of this discomfort occurring, given the more than low-risk population participating in the study.
- Psychological harms, including feelings of distress or anxiety, for example, due to disclosure of sensitive information on questionnaires and/or engaging in therapeutic activities on the app. There is a moderate likelihood and low severity of this discomfort occurring, given the more than low-risk population participating in the study.

10.2. Adverse Events or Adverse Reactions

Adverse events (AE) are considered any untoward medical occurrence in a patient or clinical trial participant administered the intervention, which does not necessarily have a causal relationship with this treatment.

AEs are assessed using the safety monitoring flow chart. Those classified as "not serious" are assessed by the research team’s qualified clinical expert. The clinical expert cannot delegate this responsibility to other research personnel.

Adverse event reports must be reported to the Coordinating Principal Investigator within 24-hours of notification. All adverse event reports must be recorded in the UNSW Safety Monitoring Register Template (Appendix D).

## 10.3. Serious Adverse Events

Serious Adverse Events (SAEs) that result in or lead to one or more of the following and the event is not related to the trial intervention:

- The death of a trial participant.
- A life-threatening illness or injury involving a trial participant.
- A participant's permanent impairment of body structure or body function.
- In-patient or prolonged hospitalisation (not for a pre-existing condition or an elective surgery) of a trial participant.
- Medical or surgical intervention to prevent life-threatening illness or injury or permanent impairment to a body structure or function of a trial participant.

SAE reports are classified following the safety assessment flowchart. SAE reports are reported to the Coordinating Principal Investigator and the Data Safety Monitoring Board (DSMB) within 24-hours. SAE reports must be recorded in the [UNSW Safety Monitoring Register Template](https://research.unsw.edu.au/document/UNSW%20Safety%20Monitoring%20Register%20Template.xlsx) (Appendix D).

## 10.4. Significant Safety Issue (SSI)

A safety issue that could adversely affect participants' safety or materially impact the trial's continued ethical acceptability or conduct. The Human Research Ethics Committee must be notified of all significant safety issues within 15 calendar days of the sponsor instigating or being made aware of the issue**.** SSI reports must be recorded in the UNSW Safety Monitoring Register Template.


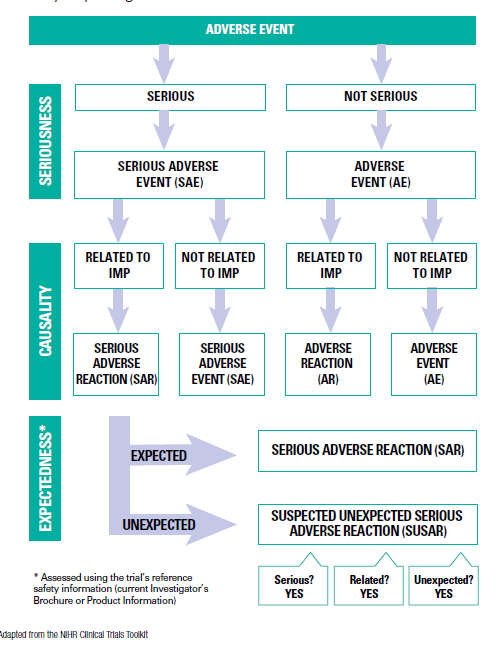


Figure 2. Safety Assessment Flow Chart Investigational Medical Product Trials

# Non-compliance, Protocol Deviation and Serious Breaches of Good Clinical Practice

## 11.1. Protocol Deviation

A protocol deviation is defined as any breach, divergence or departure from the requirements of Good Clinical Practice, the clinical trial protocol, the clinical trial standard operating procedures, or the human ethics approval that does not have a significant impact on the continued safety or rights of participants or the reliability and robustness of the data generated in the research or clinical trial. Protocol deviations are events that do not occur persistently or systematically and do not potentially result in participant harms. Examples of protocol deviations include but are not limited to:

- Deviations because of participant adherence to the protocol, including rescheduled study visits, participants refusal to complete scheduled research activities or failure to complete self-report questionnaires required by the study protocol.
- The completion of consent forms, safety monitoring report, case report forms or data collection tools in a manner that is not consistent with the protocol instructions or failure to make reports within the required reporting timeframes.
- Administration of the clinical trial investigational medical product or device in a manner that is not consistent with the manufacturer's instructions for use.
- Use of an unapproved version of the participant information statement or recruitment of participants using unapproved recruitment procedures.
- Inclusion of a participant that does not meet the inclusion criteria.
- An urgent safety measure must be taken to eliminate an immediate hazard to a participant's health or safety.

## 11.2 Serious Breach of Good Clinical Practice

A serious breach is defined as a breach of Good Clinical Practice, the clinical trial protocol, the clinical trial standard operating procedures, or the human ethics approval that is likely to affect to a significant degree the safety or rights of participants or the reliability and robustness of the data generated in the clinical trial. Examples of serious breaches include but are not limited to:

- Persistent or systematic non-compliance with the instructions for completing consent forms, safety monitoring or data collection tools that result in continued missed or incomplete data collection.
- Failure to record or report adverse events, serious adverse events, or significant safety issues where urgent safety measures were implemented.
- Widespread and uncontrolled use of protocol waivers affecting eligibility criteria, which leads to harm to trial subjects.
- Failure to conduct research following the issued approvals, permits or licences by required laws, regulations, disciplinary standards, and UNSW policies relating to the responsible or safe conduct of research.
- Concealing or facilitating breaches (or potential breaches) of the Research Code by others.
- Researching without the requisite approvals, permits or licences required by laws, regulations, disciplinary standards, and UNSW policies related to the responsible or safe conduct of research.
- Failure to conduct research as approved by an ethics review body where that conduct leads to (or has the potential to) results in participant harms.
- Researching without ethics approval as required by the National Statement on Ethical Conduct in Human Research where that conduct leads to (or has the potential to) result in participant harms.
- Any breaches as outlined in the UNSW Research Misconduct Procedure or the Australian Code for responsible conduct of research that leads to (or can potentially) result in participant harms.

## 11.3. Reporting Protocol Deviations

- Protocol deviations occurring at a site must be documented in site files and reported by the principal site investigator to the Coordinating Principal Investigator.
- The Coordinating Principal Investigator must review the protocol deviation and the clinical trial protocol to establish the corrective actions and preventative steps to prevent the deviation from reoccurring.
- The protocol deviation and corrective action plan must be reported to the UNSW Sponsor's Delegate by the Coordinating Principal Investigator or Coordinating Research Team using the protocol deviation report form.

## 11.4. Reporting of a Serious Breach

- The Coordinating Principal Investigator must review the serious breach, along with the clinical trial protocol, to develop a Corrective and Preventive Action (CAPA) that defines the steps to prevent the serious breach from reoccurring.
- The serious breach report and the CAPA must be provided to the approving HREC, and the UNSW sponsors delegate for review and approval.

## **11.5.** Review of a Protocol Deviation and a Serious Breach

- The UNSW HREC will review reports to establish whether the event meets the definition of a protocol deviation or serious breach, establish whether the proposed CAPA is appropriate and establish whether there is or will be ongoing impact reliability and robustness of the data generated.
- Protocol deviation or serious breach reports where a UNSW researcher, staff or student is responsible for the protocol deviation or the serious breach will be reviewed as per the [UNSW Research Misconduct Procedure](https://www.gs.unsw.edu.au/policy/documents/researchmisconductproc.pdf) to establish a breach of the [UNSW Research Code of Conduct](https://www.gs.unsw.edu.au/policy/documents/researchcode.pdf) has occurred.

**Table 2. Guidance for Safety and Adverse Event Reporting and Serious Breaches of Protocol**

| Type of Event | Example | How | When |
| --- | --- | --- | --- |
| Adverse Event | Complaints from the participants that indicate the negative impact of app usage on mental health | Email to the DSMB and the UNSW Human Research Ethics Committee | Within 24 business hours of becoming aware of the event |
| Investigator’s Brochure Updates/Addenda | - | Email to the UNSW Human Research Ethics Committee | Annually with the annual safety report, or as required. |
| Annual safety report | - | Annual Progress Report | Annual as required by the HREC |
| Serious Breach of Protocol | - | Email to the DSMB and the UNSW Human Research Ethics Committee | Within 24 business hours of becoming aware of the event |

# Risk management

## 12.1. Risk mitigation strategies

To minimise known harms, risks, and discomfort to participants, the below procedures will be implemented in the study. In both the control and intervention apps there is a help button that contains a directory of key sources of help such as phone numbers for Kids Helpline and LifeLine to ensure that participants have access to support 24/7. Participants are also reminded of contacting their GPs when feel distressed and maintaining their regular medicine during the trial.

In addition, there is a “get help now [panic button]” under this “help button”. When young people click the “get help now button”, a screen will pop up in the app to seek confirmation that they want to be contacted by a clinical psychologist during business hours. Once confirmed, a text message containing young people’s contact number will be sent to the clinical psychologist. They will be contacted by the clinical psychologist within the next 24 business hours.

In the middle of the trial (3-weeks after baseline assessment), young people will be asked to complete a safety check (approximately 1 minute) to assess their suicidal thoughts (using the SIDAS screener). If they report a SIDAS score above 20 they will be contacted by a clinical psychologist to refer them to appropriate psychological support, if they consent.

In addition, an independent Data Safety and Monitoring Board (DSMB) will be established to monitor the safety of research participants. DSMB membership will consist of 3 external members who are experts in clinical trial conduct, statistics, and youth mental health. The research team will record the number of notifications and notify the DSMB when each assessment period is complete. The DSMB will provide recommendations to the research team to continue, temporarily pause conduct of the trial, or discontinue, pending risk issues or adverse events. The research team will record all risk alerts in a case report which will be securely stored on the Black Dog Institute’s UNSW server.

The DSMB Charter is attached as Appendix E.

## 12.2. Privacy, confidentiality, and data management

The sensitive data collected by this study includes participants' responses to the personal detail questionnaire, their responses to the surveys, and the audio records of the interviews. This data is collected by the Black Dog Institute e-health platform. It is stored on UNSW servers (MED/BDI) and protected by encryption.

Each participant will be assigned a unique identification code automatically at the time of registration on the Black Dog Institute research platform. When online survey data is exported for analysis, the chief investigator will remove the identifiable information from this initial data set (I.e. remove first name, mobile phone number, and email address).

A de-identified copy of the data will be downloaded for analysis on the UNSW OneDrive, which is password protected and approved by the University for storing highly sensitive data. The file used for analysis will only include the unique ID code and raw research data. Only named study personnel will have access to any identifiable information. For participant withdrawal, only named study personnel will access the register to identify the personal details of participant using their ID code.

The transcription of the interviews will be undertaken by a professional transcription service, and only deidentified audio will be provided to the agency. The agency will be asked to sign a confidentiality agreement before being provided with the recordings. Participants will be informed that deidentified audio will be provided to an agency for transcription service only. Upon completion of this project, all information will be stored in an archive on the server hard drive for a period of a minimum of 7 years, in accordance with UNSW and the Australian Code for the Responsible Conduct of Research.

# References

1. Australian Bureau of Statistics, Causes of Death, no.3303.0. 2017, Australian Bureau of Statistics: Canberra.
2. Joiner, T.E., Jr., J.J. Pfaff, and J.G. Acres, A brief screening tool for suicidal symptoms in adolescents and young adults in general health settings. Behaviour research and therapy, 2002. 40(4): p. 471-81.
3. Nock, M.K., et al., Prevalence, correlates, and treatment of lifetime suicidal behavior among adolescents: results from the National Comorbidity Survey Replication Adolescent Supplement. JAMA Psychiatry, 2013. 70(3): p. 300-10.
4. Kinchin, I. and C.M. Doran, The Cost of Youth Suicide in Australia. International journal of environmental research and public health, 2018. 15(4).
5. Hom, M.A., I.H. Stanley, and T.E. Joiner, Jr., Evaluating factors and interventions that influence help-seeking and mental health service utilization among suicidal individuals: A review of the literature. Clinical psychology review, 2015. 40: p. 28-39.
6. Cheung, A.H. and C.S. Dewa, Mental health service use among adolescents and young adults with major depressive disorder and suicidality. Canadian journal of psychiatry, 2007. 52(4)
7. Christensen, H., P.J. Batterham, and B. O'Dea, E-health interventions for suicide prevention. International journal of environmental research and public health, 2014. 11(8): p. 8193-8212.
8. Witt, K., et al., Effectiveness of online and mobile telephone applications (‘apps’) for the self-management of suicidal ideation and self-harm: a systematic review and meta-analysis. BMC Psychiatry, 2017. 17(1): p. 297.
9. Twenge, J.M., G.N. Martin, and W.K. Campbell, Decreases in psychological well-being among American adolescents after 2012 and links to screen time during the rise of smartphone technology. Emotion, 2018. 18(6): p. 765-780.
10. DeCou CR, Comtois KA, Landes SJ. Dialectical behavior therapy is effective for the treatment of suicidal behavior: A meta-analysis. Behav Ther. 2019;50(1):60-72. PMID: 30661567. doi: 10.1016/j.beth.2018.03.009.
11. 7. Ougrin D, Tranah T, Stahl D, Moran P, Asarnow JR. Therapeutic Interventions for Suicide Attempts and Self-Harm in Adolescents: Systematic Review and Meta-Analysis. J Am Acad Child Psy. 2015 Feb;54(2):97-107. PMID: 25617250. doi: 10.1016/j.jaac.2014.10.009.
12. 8. Swales MA, Heard HL. Dialectical behaviour therapy: distinctive features: Taylor & Francis; 2016. ISBN: 1134834144.
13. 9. Palmier-Claus J, Taylor P, Varese F, Pratt D. Does unstable mood increase risk of suicide? Theory, research and practice. J Affect Disord. 2012;143(1-3):5-15. PMID: 22842024. doi: 10.1016/j.jad.2012.05.030.
14. 10. Bowen R, Balbuena L, Peters EM, Leuschen-Mewis C, Baetz M. The Relationship between Mood Instability and Suicidal Thoughts. Arch Suicide Res. 2015 Apr 3;19(2):161-71. PMID: 25774548. doi: 10.1080/13811118.2015.1004474.
15. 11. Klonsky ED, May A. Rethinking impulsivity in suicide. Suicide Life Threat Behav. 2010;40(6):612-9. PMID: 21198330. doi: 10.1521/suli.2010.40.6.612.
16. 12. Liu RT, Trout ZM, Hernandez EM, Cheek SM, Gerlus N. A behavioral and cognitive neuroscience perspective on impulsivity, suicide, and non-suicidal self-injury: Meta-analysis and recommendations for future research. Neurosci Biobehav Rev. 2017;83:440-50. PMID: 28928071. doi: 10.1016/j.neubiorev.2017.09.019.
17. 13. Stravynski A, Boyer R. Loneliness in relation to suicide ideation and parasuicide: A population-wide study. Suicide Life Threat Behav. 2001;31(1):32-40. PMID: 11326767. doi: 10.1521/suli.31.1.32.21312.
18. 14. Linehan MM, Armstrong HE, Suarez A, Allmon D, Heard HL. Cognitive-Behavioral Treatment of Chronically Parasuicidal Borderline Patients. Arch Gen Psychiat. 1991 Dec;48(12):1060-4. PMID: 1845222. doi: 10.1001/archpsyc.1991.01810360024003.
19. 15. Linehan MM, Korslund KE, Harned MS, Gallop RJ, Lungu A, Neacsiu AD, et al. Dialectical Behavior Therapy for High Suicide Risk in Individuals With Borderline Personality Disorder A Randomized Clinical Trial and Component Analysis. JAMA psychiatry. 2015 May;72(5):475-82. PMID: 26332352. doi: 10.1001/jamapsychiatry.2014.3039.
20. 16. McCauley E, Berk MS, Asarnow JR, Adrian M, Cohen J, Korslund K, et al. Efficacy of dialectical behavior therapy for adolescents at high risk for suicide: a randomized clinical trial. JAMA psychiatry. 2018;75(8):777-85. PMID: 29926087. doi: 10.1001/jamapsychiatry.2018.1109.
21. 17. Mehlum L, Tormoen AJ, Ramberg M, Haga E, Diep LM, Laberg S, et al. Dialectical Behavior Therapy for Adolescents With Repeated Suicidal and Self-harming Behavior: A Randomized Trial. J Am Acad Child Psy. 2014 Oct;53(10):1082-91. PMID: 25245352. doi: 10.1016/j.jaac.2014.07.003.
22. Fleming, T., et al., Beyond the Trial: Systematic Review of Real-World Uptake and Engagement With Digital Self-Help Interventions for Depression, Low Mood, or Anxiety. Journal of medical Internet research, 2018. 20(6): p. e199.
23. van Spijker, B. A., Batterham, P. J., Calear, A. L., Farrer, L., Christensen, H., Reynolds, J., & Kerkhof, A. J. (2014). The suicidal ideation attributes scale (SIDAS): Community-based validation study of a new scale for the measurement of suicidal ideation. Suicide and Life-Threatening Behavior, 44(4), 408-419. doi:10.1111/sltb.12084
24. Kroenke, K., Spitzer, R. L., & Williams, J. B. (2001). The PHQ-9: validity of a brief depression severity measure. Journal of General Internal Medicine, 16(9), 606-613.
25. Spitzer, R. L., Kroenke, K., Williams, J. B., & Lowe, B. (2006). A brief measure for assessing generalized anxiety disorder: the GAD-7. Archives of Internal Medicine, 166(10), 1092-1097.
26. Batterham, P. J., Sunderland, M., Carragher, N., Calear, A. L., Mackinnon, A. J., & Slade, T. (2016). The Distress Questionnaire-5: Population screener for psychological distress was more accurate than the K6/K10. Journal of Clinical Epidemiology, 71, 35-42. doi:10.1016/j.jclinepi.2015.10.005.
27. Fat LN, Scholes S, Boniface S, Mindell J, Stewart-Brown S. Evaluating and establishing national norms for mental wellbeing using the short Warwick–Edinburgh Mental Well-being Scale (SWEMWBS): findings from the Health Survey for England. Qual Life Res. 2017;26(5):1129-44. PMID: 27853963. doi: 10.1007/s11136-016-1454-8.
28. Tennant R, Hiller L, Fishwick R, Platt S, Joseph S, Weich S, et al. The Warwick-Edinburgh mental well-being scale (WEMWBS): development and UK validation. Health Qual Life Outcomes. 2007;5(1):63. PMID: 18042300. doi: 10.1186/1477-7525-5-63.
29. Morin, C. M., Belleville, G., Bélanger, L., & Ivers, H. (2011). The Insomnia Severity Index: psychometric indicators to detect insomnia cases and evaluate treatment response. Sleep, 34(5), 601-608. doi:10.1093/sleep/34.5.601
30. McEvoy, P. M., Thibodeau, M. A., & Asmundson, G. J. G. (2014). Trait Repetitive Negative Thinking: A Brief Transdiagnostic Assessment. Journal of Experimental Psychopathology, 5(3), 1-17. doi:10.5127/jep.037813
31. Bryan, C. J., David Rudd, M., Wertenberger, E., Etienne, N., Ray-Sannerud, B. N., Morrow, C. E., . . . Young-McCaughon, S. (2014). Improving the detection and prediction of suicidal behavior among military personnel by measuring suicidal beliefs: An evaluation of the Suicide Cognitions Scale. Journal of Affective Disorders, 159, 15-22. doi:https://doi.org/10.1016/j.jad.2014.02.021
32. Simons, J. S., & Gaher, R. M. (2005). The Distress Tolerance Scale: Development and Validation of a Self-Report Measure. Motivation and Emotion, 29(2), 83-102. doi:10.1007/s11031-005-7955-3.
33. Hughes, M. E., Waite, L. J., Hawkley, L. C., & Cacioppo, J. T. (2004). A Short Scale for Measuring Loneliness in Large Surveys: Results From Two Population-Based Studies. Research on Aging, 26(6), 655-672. doi:10.1177/0164027504268574
34. Wilson, C. J., Deane, F. P., Ciarrochi, J., & Rickwood, D. (2005). Measuring Help-Seeking Intentions: Properties of the General Help-Seeking Questionnaire. Canadian Journal of Counselling, 39(1), 15-28.
35. Beecham, J., & Knapp, M. R. J. (2001). Costing psychiatric interventions. In G. Thornicroft (Ed.), Measuring Mental Health Needs (Second Edition) (pp. 200-224). London: Royal College of Psychiatrists.
36. Duckworth, A. L., Peterson, C., Matthews, M. D., & Kelly, D. R. (2007). Grit: perseverance and passion for long-term goals. *Journal of Personality and Social Psychology, 92*(6), 1087-1101. doi:10.1037/0022-3514.92.6.1087
37. Duckworth, A. L., & Quinn, P. D. (2009). Development and Validation of the Short Grit Scale (Grit–S). *Journal of Personality Assessment, 91*(2), 166-174.
38. Buri, J. R., Cromett, C. E., Post, M. C., Landis, A. M., & Alliegro, M. C. (2015). Negative Life Events Scale for Students (NLESS). Paper presented at the 123rd annual convention of the American Psychological Association, Toronto, Canada.
39. O’Dea B, Han J, Batterham PJ, Achilles MR, Calear AL, Werner‐Seidler A, et al. A randomised controlled trial of a relationship-focussed mobile phone application for improving adolescents’ mental health. J Child Psychol Psychiatry. 2020. PMID: 32683737. doi: 10.1111/jcpp.13294
40. Ritchie, J. and L. Spencer, Qualitative data analysis for applied policy research, in Analyzing Qualitative Data, A. Bryman and B. Burgess, Editors. 2002, Routledge: London. p. 173-194
41. Anderson, K., Burford, O., & Emmerton, L. (2016). Mobile Health Apps to Facilitate Self- Care: A Qualitative Study of User Experiences. PloS One, 11(5), e0156164. doi:10.1371/journal.pone.0156164
42. Asklund, I., Samuelsson, E., Hamberg, K., Umefjord, G., & Sjöström, M. (2019). User Experience of an App-Based Treatment for Stress Urinary Incontinence: Qualitative Interview Study. Journal of Medical Internet Research, 21(3), e11296. doi:10.2196/11296
43. Fuller-Tyszkiewicz, M., Richardson, B., Klein, B., Skouteris, H., Christensen, H., Austin, D., . . . Ware, A. (2018). A Mobile App–Based Intervention for Depression: End-User and Expert Usability Testing Study. JMIR Ment Health, 5(3), e54. doi:10.2196/mental.9445

# Appendix A: Recruitment materials

**The LifeBuoy App: A randomised controlled trial of a mHealth intervention to help young people manage suicidal thoughts**

We are looking for young people who have experienced suicidal thoughts to participate in a study evaluating the effectiveness of a smartphone app “LifeBuoy” that is designed to reduce suicidal thoughts.

We need young people who:

- Are 16-25 years old;
- Are living in Australia;
- Are fluent in English;
- Own and have access to a smartphone;
- Have experienced suicidal thoughts in the past year;
- Have not been diagnosed with psychosis or a bipolar disorder;
- Have no suicidal attempt or plan in the past month.

Eligible participants will:

1. Be assigned to one of two groups: an intervention group (who receives the therapeutic app) or a control group (who receives a non-therapeutic app). All participants will then follow the same process;
2. Complete 7 modules in the app over 6 weeks (total 1 hour);
3. Complete an online survey on 3 occasions: before (35 minutes) and after completing all modules (25 minutes), and then 3 months after the completion of all modules (25 minutes). The survey will include questions about your demographics, suicidal thoughts and suicide attempts, negative life events, how you have been feeling lately, sleep, help-seeking intentions and experience, perseverance, and expectations of and satisfaction with the app;
4. Download an app called ‘Lifebuoy’ after completing the first survey, (you will not know whether you are receiving the intervention or control version of this app). Both apps will be available for 6 weeks for use on your personal smartphone. If you receive the control app, you will be informed after the final survey to let you know that you were in the control condition, and what that means.
5. Receive a $20 gift card in acknowledgement of your time and internet expenses related to participation in each follow-up survey (total $40 gift card if you complete both follow-up surveys).

Please note: suicide related questions will be asked in the online survey. Participants are free to withdraw at any time. The identified information obtained in this study will remain confidential and be stored in an archive on the UNSW server hard drive for a period of 7 years.

If you are interested in participating this study, please click here <Insert link: XXX>.

This study has been approved by Human Review Ethics Committee at the University of New South Wales [HC190764]

**Advertisement for social media channels**

The Black Dog Institute is inviting young people aged 18-25 who have experienced suicidal thoughts to participate in a study evaluating the effectiveness of a smartphone app designed to reduce suicidal thoughts. You will receive $40 in appreciation for your time in participating. This study involves the completion of 7 app modules over 6 weeks (1 hour total), and one online survey before starting the app (35 minutes) and two surveys after (immediately and 3-months later; 25 minutes each).

Click here <Insert the link to the recruitment page> to get more information. Lifeline: 13 11 14.

**Short version of the advertisement**

The Black Dog Institute is inviting young people aged between 16-25 who have experienced suicidal thoughts to evaluate the effectiveness of a smartphone app designed to reduce suicidal thoughts. You will receive $40 in giftcards for participating.

Click here<Insert the link to the recruitment page> to know more. Lifeline: 13 11

# Appendix B: Screening and Eligibility

**Screening questions**

| **What the participant will see** | | **Drop down response options available** |
| --- | --- | --- |
| Are you aged between 16 and 25? | --- select --- | Yes, I’m between 16 and 20 years old  Yes, I’m between 21 and 25 years old  No |
| Are you living in Australia currently? | --- select --- | Yes  No |
| Are you fluent in English? | --- select --- | Yes  No |
| Do you own, or have access to, a smartphone (iOS or Android)? | -- select --- | Yes  No |
| Have you experienced suicidal thoughts in the past 12 months? | -- select --- | Yes  No |
| Have you attempted suicide in the past month? | -- select --- | Yes  No |
| Have you ever been diagnosed with psychosis or bipolar disorder? | -- select --- | Yes  No |
| Gender assigned at birth | -- select --- | Male  Female |

**Wording following screening outcomes**

**(If eligible)**

Ok great, thank you so much for taking the time to do that.

Based on your responses, it looks like you are:

| **Eligible!** |
| --- |

That is great news! If you are willing to take part in the study, we will need to click through to the next page and read our participant information sheet and provide consent. This will help you make a decision about whether you do want to participate or not, so please read all information carefully.

Please remember you do not have to participate if you don’t want to, and you can withdraw your consent at any time.

If you’ve had a tough time with your mental health lately, we encourage you to talk to a local doctor or mental health professional about how you’re feeling.

If you would like to know where to get some immediate help, here are some suggestions:

Lifeline: 13 11 14

Kids Helpline: 1800 55 1800

Suicide Callback Service: 1300 659 467

For 24 hour advice on what to do and who to contact, don’t hesitate to visit HealthDirect

( <https://www.healthdirect.gov.au/>) or call them on 1800 022 222.

They can help you figure out the best people to contact in your local area

**If you feel in danger or that you might hurt yourself, do not hesitate to call Emergency Services on 000.**

(**If ineligible)**

Ok great, thank you for your interest in our study.

Based on your responses, it looks like you are:

| **Ineligible** |
| --- |

Thank you for your interest in our study.

Based on your responses, your participation in this study is now complete.

[response 1] Your responses indicate that you are unable to participate in this study because you don’t meet the selection criteria.

[response 2] Your responses indicate that now is not the best time for you to participate in this study because participating at this time might cause you to become distressed, or uncomfortable.

Your willingness to participate in this study is hugely appreciated. And just because this might not be the right study for you, or the right time for you to participate, it does not mean you are on your own.

If you’ve had a tough time with your mental health lately, we encourage you to talk to a local doctor or mental health professional about how you’re feeling.

If you would like to know where to get some immediate help, here are some suggestions:

**Lifeline:** 13 11 14

**Kids Helpline:** 1800 55 1800

**Suicide Callback Service:** 1300 659 467

For 24 hour advice on what to do and who to contact, don’t hesitate to visit HealthDirect

( https://www.healthdirect.gov.au/ ) or call them on 1800 022 222.

They can help you figure out the best people to contact in your local area.

If you feel in danger or that you might hurt yourself, do not hesitate to call Emergency Services on 000.

# Appendix C: Survey Measures

| **What the participant will see** | | **Drop down response options available** |
| --- | --- | --- |
| Age | --- select ---- | 16  17  18  19  20  21  22  23  24  25 |
| Gender identity | --- select ---- | Female  Male  Non-binary  Different identity |
| Gender assigned at birth | --- select ---- | Female  Male |
| Where do you live? | -- select ---- | NSW  QLD  VIC  TAS  SA  WA  NT  ACT |
| Rural/remote or metropolitan | -- select ---- | Rural and/or remote  Metropolitan |
| Do you identify as being Lesbian, Gay, Bisexual, Transgender, or Intersex? | -- select ---- | No  Lesbian  Gay  Bisexual  Transgender  Intersex  Prefer not to say |
| Indigenous status | -- select ---- | Not Aboriginal or Torres Strait Islander  Yes - Torres Strait Islander Yes – Aboriginal  I prefer not to say |
| Language/s spoken at home | -- select ---- | English only  English and other language  Another language only |
| Current living situation | -- select ---- | Live alone  Live with parent/s/family,  Live with a significant other  Live with roommate(s)  Other |
| Current relationship situation | -- select ---- | Not in a relationship  Boyfriend/Girlfriend  De facto/married Separated  Divorced or Widowed  Other |
| Highest level of education | -- select ---- | Primary school  Years 7 to 9  Year 10 or equivalent  Year 11  Year 12 or equivalent  Certificate Level I - IV Diploma/Associate Degree  Graduate Diploma/Certificate  Bachelor Degree  Master degree  Doctoral degree |
| What is your current employment status? | -- select ---- | Full-time  Part-time  Casual  Self-employed  Unemployed |
| Has your current employment status changed as a result of the coronavirus (COVID-19) pandemic? | --- select ---- | Yes  No |
| Have you ever experienced mental illness or been diagnosed with mental illness? | -- select ---- | Yes  No  I don’t know. |
| Have you ever seen a mental health professional (psychologist, psychiatrist) for a mental health problem? | --- select ---- | Yes  No  I don’t know. |
| Have you previously used any health or wellbeing apps on your smartphone? | --- select ---- | Yes  No  I don’t know. |

**Suicidal Ideation Attributes Scale (SIDAS)**

**Now we’re going to ask you some questions are about your suicidal thoughts, and the severity of them.**

**Please think about the past month when answering the following questions.**

##

| In the past month, how often have you had thoughts about suicide? | | | | | | | | | | |
| --- | --- | --- | --- | --- | --- | --- | --- | --- | --- | --- |
| 0  Never | 1 | 2 | 3 | 4 | 5 | 6 | 7 | 8 | 9 | 10  Always |
| In the past month, how much control have you had over these thoughts? | | | | | | | | | | |
| 0  No control | 1 | 2 | 3 | 4 | 5 | 6 | 7 | 8 | 9 | 10  Full control |
| In the past month, how close have you come to making a suicide attempt? | | | | | | | | | | |
| 0  Not close | 1 | 2 | 3 | 4 | 5 | 6 | 7 | 8 | 9 | 10  Made an attempt |
| In the past month, to what extent have you felt tormented by thoughts about suicide? | | | | | | | | | | |
| 0  Not at all | 1 | 2 | 3 | 4 | 5 | 6 | 7 | 8 | 9 | 10  Extremely |
| In the past month, how much have thoughts about suicide interfered with your ability to carry out daily activities, such as work, household tasks or social activities? | | | | | | | | | | |
| 0  Not at all | 1 | 2 | 3 | 4 | 5 | 6 | 7 | 8 | 9 | 10  Extremely |

**We’d just like to ask you a few more questions about prior self-harm behaviour. Just to remind you, these questions are completely voluntary.**

| **What the user will see** | | **Drop down response options available** |
| --- | --- | --- |
| Have you ever attempted suicide? | --- select ---- | No, never Yes, once  Yes, more than once |
| If more than once, how many times? | --- select ---- | 2  3  4  5  6+ |
| How many months have you been thinking about suicide? | --- select ---- | 1 or less  2  3 to 6  >6 to 12  >12 to 24  >24 to 36  >36 |
| How often, on average, do you think about suicide? | --- select ---- | Daily Weekly Monthly  Less than monthly |
| Have you attempted suicide in the past 30 days? | --- select ---- | No, never Yes, once  Yes, more than once |
| Have you ever injured yourself on purpose (i.e., intentional, self-inflicted damage to the surface of the body without suicidal intent) | --- select ---- | No, never  Yes, once  Yes, more than once |
| In the past month, how often have you injured yourself on purpose? | - If ‘Yes, more than once’ then select - | 2-5 times (low)  6-9 times (moderate)  10-20 times (high)  20+ times (severe) |
| Please rate how serious your worst injury was in the past month | - If ‘Yes, once’ or ‘yes, more than once’ then select - | No care was needed (mild)  Some care was needed (moderate)  I required medical care (severe) |

| If you are feeling distressed, please contact any of the following numbers |
| --- |
| Lifeline  13 11 14 |
| Kids Helpline  1800 55 1800 |
| Suicide Callback Service  1300 659 467 |

**Patient Health Questionnaire-9 (PHQ-9)**

**Over the past 2 weeks, how often have you been bothered by the following problems?**

| Little interest or pleasure in doing things | | | |
| --- | --- | --- | --- |
| Not at all | Several days | More than half the days | Nearly every day |
| Feeling down, depressed, irritable, or hopeless | | | |
| Not at all | Several days | More than half the days | Nearly every day |
| Trouble falling asleep, staying asleep, or sleeping too much | | | |
| Not at all | Several days | More than half the days | Nearly every day |
| Feeling tired, or having little energy | | | |
| Not at all | Several days | More than half the days | Nearly every day |
| Poor appetite, weight loss, or overeating | | | |
| Not at all | Several days | More than half the days | Nearly every day |
| Feeling bad about yourself — or feeling that you are a failure, or that you have let yourself or your family down | | | |
| Not at all | Several days | More than half the days | Nearly every day |
| Trouble concentrating on things, such as reading the newspaper or watching television | | | |
| Not at all | Several days | More than half the days | Nearly every day |
| Moving or speaking so slowly that other people could have noticed? Or the opposite — being so fidgety or restless that you were moving around a lot more than usual | | | |
| Not at all | Several days | More than half the days | Nearly every day |
| Thoughts that you would be better off dead, or of hurting yourself in some way | | | |
| Not at all | Several days | More than half the days | Nearly every day |

**Distress Questionnaire-5 (DQ-5)**

**Thinking about the last 30 days, how often were you bothered by the following problems? Again, please click on only one answer per line.**

| My worries overwhelmed me | | | | |
| --- | --- | --- | --- | --- |
| Never | Rarely | Sometimes | Often | Always |
| I felt hopeless | | | | |
| Never | Rarely | Sometimes | Often | Always |
| I found social settings upsetting | | | | |
| Never | Rarely | Sometimes | Often | Always |
| I had trouble staying focused on tasks | | | | |
| Never | Rarely | Sometimes | Often | Always |
| Anxiety or fear interfered with my ability to do the things I needed to do at work or at home | | | | |
| Never | Rarely | Sometimes | Often | Always |

**Repetitive Think Questionnaire (RTQ-10)**

**For the next part, please take a minute to think about a recent event where you did something that you found distressing. How true are each of these statements with respect to that experience.**

| You had thoughts or images about all your shortcomings, failings, faults, mistakes. | | | | |
| --- | --- | --- | --- | --- |
| 1  Not true at all | 2 | 3  Somewhat true | 4 | 5  Very true |
| You had thoughts or images about a past event that came into your head even when you did not wish to think about it again | | | | |
| 1  Not true at all | 2 | 3  Somewhat true | 4 | 5  Very true |
| You had thoughts or images that “I won’t be able to do my job/work because I feel so badly.” | | | | |
| 1  Not true at all | 2 | 3  Somewhat true | 4 | 5  Very true |
| You had thoughts or images of the situation that were difficult to forget. | | | | |
| 1  Not true at all | 2 | 3  Somewhat true | 4 | 5  Very true |
| Once I started thinking about the situation, I couldn’t stop | | | | |
| 1  Not true at all | 2 | 3  Somewhat true | 4 | 5  Very true |
| I noticed that I had been thinking about the situation. | | | | |
| 1  Not true at all | 2 | 3  Somewhat true | 4 | 5  Very true |
| You had thoughts or images of the situation that you tried to resist thinking about. | | | | |
| 1  Not true at all | 2 | 3  Somewhat true | 4 | 5  Very true |
| I thought about the situation all the time. | | | | |
| 1  Not true at all | 2 | 3  Somewhat true | 4 | 5  Very true |
| I knew I shouldn’t have thought about the situation, but I couldn’t help it | | | | |
| 1  Not true at all | 2 | 3  Somewhat true | 4 | 5  Very true |
| You had thoughts or images about the situation and wishing it had gone better. | | | | |
| 1  Not true at all | 2 | 3  Somewhat true | 4 | 5  Very true |

**Suicide Cognitions Scale (SCS)**

**The next questions are about you and your suicidal thoughts. These may represent potential things you think about which could affect the severity of your thoughts. Please rank each item on how much you agree or disagree with the statement.**

| No one can help me solve my problems | | | | |
| --- | --- | --- | --- | --- |
| Disagree strongly | Disagree | Neither agree or disagree | Agree | Agree strongly |
| I am completely unworthy of love | | | | |
| Disagree strongly | Disagree | Neither agree or disagree | Agree | Agree strongly |
| It is impossible to describe how badly I feel | | | | |
| Disagree strongly | Disagree | Neither agree or disagree | Agree | Agree strongly |
| I can’t cope with my problems any longer | | | | |
| Disagree strongly | Disagree | Neither agree or disagree | Agree | Agree strongly |
| I can’t imagine anyone being able to withstand this kind of pain | | | | |
| Disagree strongly | Disagree | Neither agree or disagree | Agree | Agree strongly |
| There is nothing redeeming about me | | | | |
| Disagree strongly | Disagree | Neither agree or disagree | Agree | Agree strongly |
| Nothing can help solve my problems | | | | |
| Disagree strongly | Disagree | Neither agree or disagree | Agree | Agree strongly |
| I don’t deserve to live another moment | | | | |
| Disagree strongly | Disagree | Neither agree or disagree | Agree | Agree strongly |
| No one is as loathsome as me | | | | |
| Disagree strongly | Disagree | Neither agree or disagree | Agree | Agree strongly |

Insomnia Severity Index (ISI)

**The next questions are about sleep difficulties. Please take a moment to think about the severity of any sleep problems you may have been having in the past 2 weeks, and then click one answer per line.**

| Difficulty falling asleep | | | | |
| --- | --- | --- | --- | --- |
| 0  None | 1 | 2  Moderate | 3 | 4  Very severe |
| Difficulty staying asleep | | | | |
| 0  None | 1 | 2  Moderate | 3 | 4  Very severe |
| Problems waking up too early | | | | |
| 0  None | 1 | 2  Moderate | 3 | 4  Very severe |
| How satisfied/dissatisfied are you with your current sleep pattern? | | | | |
| 0  Very satisfied | 1 | 2  Moderately satisfied | 3 | 4  Very dissatisfied |
| How noticeable to others do you think your sleep problem is in terms of impairing the quality of your life? | | | | |
| 0  Not noticeable | 1 | 2  Somewhat | 3 | 4  Very noticeable |
| How worried are you about your current sleep problem? | | | | |
| 0  Not worried | 1 | 2  Somewhat | 3 | 4  Very worried |
| To what extent do you consider your sleep problems to interfere with your daily functioning (e.g. mood, concentration, memory, mood, etc)? | | | | |
| 0  Not interfering | 1 | 2  Somewhat | 3 | 4  Very interfering |

Short Warwick Edinburgh Mental Wellbeing Scale (SWEMWBS)

**Below are some statements about feelings and thoughts. Please click one answer pre question that best describes your experience of each over the last 2 weeks.**

| I’ve been feeling optimistic about the future. | | | | |
| --- | --- | --- | --- | --- |
| None of the time | Rarely | Some of the time | Often | All of the time |
| I’ve been feeling useful. | | | | |
| None of the time | Rarely | Some of the time | Often | All of the time |
| I’ve been feeling relaxed. | | | | |
| None of the time | Rarely | Some of the time | Often | All of the time |
| I’ve been dealing with problems well. | | | | |
| None of the time | Rarely | Some of the time | Often | All of the time |
| I’ve been thinking clearly. | | | | |
| None of the time | Rarely | Some of the time | Often | All of the time |
| I’ve been feeling close to other people. | | | | |
| None of the time | Rarely | Some of the time | Often | All of the time |
| I’ve been able to make up my own mind about things. | | | | |
| None of the time | Rarely | Some of the time | Often | All of the time |

**Generalized Anxiety Disorder Questionnaire (GAD-7)**

**Over the past 2 weeks, how often have you been bothered by the following problems?**

| Feeling nervous, anxious, or on edge | | | |
| --- | --- | --- | --- |
| Not at all | Several days | More than half the days | Nearly every day |
| Not being able to stop or control worrying | | | |
| Not at all | Several days | More than half the days | Nearly every day |
| Worrying too much about different things | | | |
| Not at all | Several days | More than half the days | Nearly every day |
| Trouble relaxing | | | |
| Not at all | Several days | More than half the days | Nearly every day |
| Being so restless that it's hard to sit still | | | |
| Not at all | Several days | More than half the days | Nearly every day |
| Becoming easily annoyed or irritable | | | |
| Not at all | Several days | More than half the days | Nearly every day |
| Feeling afraid as if something awful might happen | | | |
| Not at all | Several days | More than half the days | Nearly every day |

**Distress Tolerance Scale (DTS)**

**Think of times that you feel distressed or upset. Select one answer per line that best describes your beliefs about feeling distressed or upset.**

| Feeling distressed or upset is unbearable to me | | | | |
| --- | --- | --- | --- | --- |
| Strongly disagree | Mildly disagree | Agree and disagree equally | Mildly agree | Strongly agree |
| When I feel distressed or upset, all I can think about is how bad I feel | | | | |
| Strongly disagree | Mildly disagree | Agree and disagree equally | Mildly agree | Strongly agree |
| I can’t handle feeling distressed or upset. | | | | |
| Strongly disagree | Mildly disagree | Agree and disagree equally | Mildly agree | Strongly agree |
| My feelings of distress are so intense that they completely take over. | | | | |
| Strongly disagree | Mildly disagree | Agree and disagree equally | Mildly agree | Strongly agree |
| There’s nothing worse than feeling distressed or upset. | | | | |
| Strongly disagree | Mildly disagree | Agree and disagree equally | Mildly agree | Strongly agree |
| I can tolerate being distressed or upset as well as most people. | | | | |
| Strongly disagree | Mildly disagree | Agree and disagree equally | Mildly agree | Strongly agree |
| My feelings of distress or being upset are not acceptable. | | | | |
| Strongly disagree | Mildly disagree | Agree and disagree equally | Mildly agree | Strongly agree |
| I’ll do anything to avoid feeling distressed or upset. | | | | |
| Strongly disagree | Mildly disagree | Agree and disagree equally | Mildly agree | Strongly agree |
| Other people seem to be able to tolerate feeling distressed or upset better than I can. | | | | |
| Strongly disagree | Mildly disagree | Agree and disagree equally | Mildly agree | Strongly agree |
| Being distressed or upset is always a major ordeal for me | | | | |
| Strongly disagree | Mildly disagree | Agree and disagree equally | Mildly agree | Strongly agree |
| I am ashamed of myself when I feel distressed or upset | | | | |
| Strongly disagree | Mildly disagree | Agree and disagree equally | Mildly agree | Strongly agree |
| My feelings of distress or being upset scare me. | | | | |
| Strongly disagree | Mildly disagree | Agree and disagree equally | Mildly agree | Strongly agree |
| I’ll do anything to stop feeling distressed or upset | | | | |
| Strongly disagree | Mildly disagree | Agree and disagree equally | Mildly agree | Strongly agree |
| When I feel distressed or upset, I must do something about it immediately | | | | |
| Strongly disagree | Mildly disagree | Agree and disagree equally | Mildly agree | Strongly agree |
| When I feel distressed or upset, I cannot help but concentrate on how bad the distress actually feels | | | | |
| Strongly disagree | Mildly disagree | Agree and disagree equally | Mildly agree | Strongly agree |

**3-item Loneliness Scale**

**The next questions are about how you feel about different aspects of your life. For each one, tell me how often you feel that way.**

| How often do you feel that you lack companionship? | | |
| --- | --- | --- |
| Hardly ever | Some of the time | Often |
| How often do you feel left out? | | |
| Hardly ever | Some of the time | Often |
| How often do you feel isolated from others | | |
| Hardly ever | Some of the time | Often |

**Short Grit Scale (SGS)**

Here are a number of statements that may or may not apply to you. When responding, think of how you compare to most people -- not just the people you know well, but most people in the world. There are no right or wrong answers, so just try your best to answer honestly.

| New ideas and projects sometimes distract me from previous ones | | | | |
| --- | --- | --- | --- | --- |
| Not like me at all | Not much like me | Somewhat like me | Mostly like me | Very much like me |
| Setbacks don’t discourage me | | | | |
| Not like me at all | Not much like me | Somewhat like me | Mostly like me | Very much like me |
| I have been obsessed with a certain idea or project for a short time but later lost interest. | | | | |
| Not like me at all | Not much like me | Somewhat like me | Mostly like me | Very much like me |
| I am a hard worker | | | | |
| Not like me at all | Not much like me | Somewhat like me | Mostly like me | Very much like me |
| I often set a goal but later choose to pursue a different one | | | | |
| Not like me at all | Not much like me | Somewhat like me | Mostly like me | Very much like me |
| I have difficulty maintaining my focus on projects that take more than a few months to complete | | | | |
| Not like me at all | Not much like me | Somewhat like me | Mostly like me | Very much like me |
| I finish whatever I begin | | | | |
| Not like me at all | Not much like me | Somewhat like me | Mostly like me | Very much like me |
| I am diligent | | | | |
| Not like me at all | Not much like me | Somewhat like me | Mostly like me | Very much like me |

**Modified-Negative Life Events Scale for Students (NLESS)**

**Below are some negative life events that have been found to produce stress or distress in young people’s lives.**

**Thinking about the past year, please let us know if these events have happened to you, and if they have, how stressful they have been (from not stressful to extremely stressful).**

| **What the participant will see** | | **Drop down response options available** |
| --- | --- | --- |
| Death of family member | --- select ---- | No, hasn’t happened  Yes, but wasn’t stressful  Yes, and slightly stressful  Yes, and moderately stressful  Yes, and definitely stressful  Yes, and extremely stressful |
| Death of close friend | --- select ---- | No, hasn’t happened  Yes, but wasn’t stressful  Yes, and slightly stressful  Yes, and moderately stressful  Yes, and definitely stressful  Yes, and extremely stressful |
| Serious illness / injury to family member | --- select ---- | No, hasn’t happened  Yes, but wasn’t stressful  Yes, and slightly stressful  Yes, and moderately stressful  Yes, and definitely stressful  Yes, and extremely stressful |
| Serious illness / injury to you | --- select ---- | No, hasn’t happened  Yes, but wasn’t stressful  Yes, and slightly stressful  Yes, and moderately stressful  Yes, and definitely stressful  Yes, and extremely stressful |
| Serious illness / injury to close friend | --- select ---- | No, hasn’t happened  Yes, but wasn’t stressful  Yes, and slightly stressful  Yes, and moderately stressful  Yes, and definitely stressful  Yes, and extremely stressful |
| Divorce of parents | --- select ---- | No, hasn’t happened  Yes, but wasn’t stressful  Yes, and slightly stressful  Yes, and moderately stressful  Yes, and definitely stressful  Yes, and extremely stressful |
| Family member arrested | --- select ---- | No, hasn’t happened  Yes, but wasn’t stressful  Yes, and slightly stressful  Yes, and moderately stressful  Yes, and definitely stressful  Yes, and extremely stressful |
| You arrested | --- select ---- | No, hasn’t happened  Yes, but wasn’t stressful  Yes, and slightly stressful  Yes, and moderately stressful  Yes, and definitely stressful  Yes, and extremely stressful |
| You having been assaulted | --- select ---- | No, hasn’t happened  Yes, but wasn’t stressful  Yes, and slightly stressful  Yes, and moderately stressful  Yes, and definitely stressful  Yes, and extremely stressful |
| Parent laid off work |  | No, hasn’t happened  Yes, but wasn’t stressful  Yes, and slightly stressful  Yes, and moderately stressful  Yes, and definitely stressful  Yes, and extremely stressful |
| Serious break-up with boyfriend / girlfriend | --- select ---- | No, hasn’t happened  Yes, but wasn’t stressful  Yes, and slightly stressful  Yes, and moderately stressful  Yes, and definitely stressful  Yes, and extremely stressful |
| Family has major financial pressures | --- select ---- | No, hasn’t happened  Yes, but wasn’t stressful  Yes, and slightly stressful  Yes, and moderately stressful  Yes, and definitely stressful  Yes, and extremely stressful |
| You had major financial pressures | --- select ---- | No, hasn’t happened  Yes, but wasn’t stressful  Yes, and slightly stressful  Yes, and moderately stressful  Yes, and definitely stressful  Yes, and extremely stressful |
| Addiction/psychological struggle of family member | --- select ---- | No, hasn’t happened  Yes, but wasn’t stressful  Yes, and slightly stressful  Yes, and moderately stressful  Yes, and definitely stressful  Yes, and extremely stressful |
| You struggled with addiction/psychological problem | --- select ---- | No, hasn’t happened  Yes, but wasn’t stressful  Yes, and slightly stressful  Yes, and moderately stressful  Yes, and definitely stressful  Yes, and extremely stressful |
| Cheated on by boyfriend/girlfriend | --- select ---- | No, hasn’t happened  Yes, but wasn’t stressful  Yes, and slightly stressful  Yes, and moderately stressful  Yes, and definitely stressful  Yes, and extremely stressful |
| Serious academic problems | --- select ---- | No, hasn’t happened  Yes, but wasn’t stressful  Yes, and slightly stressful  Yes, and moderately stressful  Yes, and definitely stressful  Yes, and extremely stressful |
| Parents have ongoing conflicts | --- select ---- | No, hasn’t happened  Yes, but wasn’t stressful  Yes, and slightly stressful  Yes, and moderately stressful  Yes, and definitely stressful  Yes, and extremely stressful |
| You have ongoing conflict with parents | --- select ---- | No, hasn’t happened  Yes, but wasn’t stressful  Yes, and slightly stressful  Yes, and moderately stressful  Yes, and definitely stressful  Yes, and extremely stressful |
| You experienced abuse/violence at home | --- select ---- | No, hasn’t happened  Yes, but wasn’t stressful  Yes, and slightly stressful  Yes, and moderately stressful  Yes, and definitely stressful  Yes, and extremely stressful |
| Family lost house through fire, flood, etc. | --- select ---- | No, hasn’t happened  Yes, but wasn’t stressful  Yes, and slightly stressful  Yes, and moderately stressful  Yes, and definitely stressful  Yes, and extremely stressful |
| Unwanted sexual behaviour imposed on you | --- select ---- | No, hasn’t happened  Yes, but wasn’t stressful  Yes, and slightly stressful  Yes, and moderately stressful  Yes, and definitely stressful  Yes, and extremely stressful |
| Unwanted pregnancy | --- select ---- | No, hasn’t happened  Yes, but wasn’t stressful  Yes, and slightly stressful  Yes, and moderately stressful  Yes, and definitely stressful  Yes, and extremely stressful |
| Serious conflict with close friend | --- select ---- | No, hasn’t happened  Yes, but wasn’t stressful  Yes, and slightly stressful  Yes, and moderately stressful  Yes, and definitely stressful  Yes, and extremely stressful |
| Serious conflict with boss at work | --- select ---- | No, hasn’t happened  Yes, but wasn’t stressful  Yes, and slightly stressful  Yes, and moderately stressful  Yes, and definitely stressful  Yes, and extremely stressful |

**General Help-Seeking Questionnaire**

Now we’re going to ask you about where you may, or may not, seek help from for suicidal thoughts. If you were having suicidal thoughts how likely is it that you would seek help from the following sources.

| A boyfriend or girlfriend | | | | | |
| --- | --- | --- | --- | --- | --- |
| Not applicable | Extremely unlikely | Unlikely | Neither | Likely | Extremely likely |
| A friend | | | | | |
| Not applicable | Extremely unlikely | Unlikely | Neither | Likely | Extremely likely |
| Parents | | | | | |
| Not applicable | Extremely unlikely | Unlikely | Neither | Likely | Extremely likely |
| Other relative/family member (e.g. brothers, sisters, aunts/uncles, grandparents) | | | | | |
| Not applicable | Extremely unlikely | Unlikely | Neither | Likely | Extremely likely |
| School or university counsellor | | | | | |
| Not applicable | Extremely unlikely | Unlikely | Neither | Likely | Extremely likely |
| Mental health professional (e.g. psychologist, psychiatrist) | | | | | |
| Not applicable | Extremely unlikely | Unlikely | Neither | Likely | Extremely likely |
| Doctor/GP | | | | | |
| Not applicable | Extremely unlikely | Unlikely | Neither | Likely | Extremely likely |
| Phone helpline (e.g. Lifeline) | | | | | |
| Not applicable | Extremely unlikely | Unlikely | Neither | Likely | Extremely likely |
| Internet website on mental health (e.g. Reachout) | | | | | |
| Not applicable | Extremely unlikely | Unlikely | Neither | Likely | Extremely likely |
| Mobile app | | | | | |
| Not applicable | Extremely unlikely | Unlikely | Neither | Likely | Extremely likely |
| I would not seek help from anyone | | | | | |
| Not applicable | Extremely unlikely | Unlikely | Neither | Likely | Extremely likely |
| I would seek help from another not listed above | | | | | |
| Not applicable | Extremely unlikely | Unlikely | Neither | Likely | Extremely likely |

**Modified-Client Service Receipt Inventory (CSRI)**

Almost done. We’d just like to ask you about some of the services you may have used in the past 6 months for mental health issues or suicidal thoughts.

| **What the participant will see** | | **Drop down response options available** |
| --- | --- | --- |
| In the last 6 months, have you used, or had contact with, any of the following **for mental health problems including suicidal thoughts**? | | |
| Hospital services | --- select ---- | yes  no |
| Mental Health helpline (e.g., LifeLine) | --- select ---- | yes  no |
| Psychiatric crisis support team | --- select ---- | Yes  no |
| Police/ambulance | --- select ---- | yes  no |
| Social worker | --- select ---- | yes  no |
| Counsellor | --- select ---- | yes  no |
| Psychologist / therapist | --- select ---- | yes  no |
| Self-help group | --- select ---- | yes  no |
| Psychiatrist | --- select ---- | yes  no |
| GP |  |  |
| Other medically qualified doctor | --- select ---- | yes  no |
| Other | --- select ---- | yes  no |

**Expectations of Success**

**We’d just like to ask you about your expectations of success of the app you’ll be trialling shortly – which is known as LifeBuoy.**

| **What the participant will see** | | **Drop down response options available** |
| --- | --- | --- |
| I am confident that people could reduce their suicidal thoughts using an app. | --- select ---- | Strongly agree  Agree  Neither agree nor disagree Disagree  Strongly disagree |
| I think that participating in a study that aims to reduce suicidal thoughts is an important thing to do. | --- select ---- | Strongly agree  Agree  Neither agree nor disagree Disagree  Strongly disagree |
| Please rate the importance of reducing your suicidal  thoughts over the next 6 months. | --- select ---- | Not important  Somewhat important Moderately important Important  Very important |
| Please rate your readiness to reduce your suicidal thoughts by using an app. | --- select ---- | Not ready  Moderately ready  Ready  Completely ready |

Thank you so much for completing this questionnaire, we greatly appreciate it.

# Appendix D: UNSW Safety Monitor Register Template

| **Section 1: Project Details** | | | | | | |
| --- | --- | --- | --- | --- | --- | --- |
| 1. **HREC/HREAP reference number:** |  | | | | | |
| 1. **Project title:** |  | | | | | |
| 1. **Chief Investigator** |  | | | | | |
| 1. **Approving HREC:** |  | | | | | |
| 1. **Type of report** | **Initial**  **Follow up** | | | | | |
| **Section 2: Details of the event** | | | | | | |
| 1. **Date of occurrence** |  | | | | | |
| 1. **Location of occurrence** |  | | | | | |
| 1. **Has the event or incident been resolved** | **Yes**  **No** | | | | | |
| 1. **Who was affected by the event or incident** | **Party Affected** | **Yes** | **No** | **If yes, provide further detail such as number of participants/records, names of researchers etc.** | | |
|  | Research Participants |  |  |  | | |
|  | Researchers |  |  |  | | |
|  | Research Records, Data or Property |  |  |  | | |
| 1. **Did the event result in or cause any of the following?** | - Death - Life-threatening - Hospitalisation - Prolongation of existing hospitalisation - persistent or significant disability or incapacity - congenital anomaly or birth defect | | | | | |
| 1. **Describe the incident using lay language. Include details of any negative consequences, harm or damage that has occurred because of the incident.** | | | | | | |
| 1. **What has been identified as the cause of the incident?** | | | | | | |
|  | | | | | | |
| 1. **Describe the corrective steps that have occurred and those that are to occur following this report.** | | | | | | |
|  | | | | | | |
| 1. **Describe the preventative steps to stop reoccurrence.** | | | | | | |
|  | | | | | | |
| 1. **Has the event/incident had an impact on the ethical acceptability of the research** | | | | | **Yes** | **No** |
| 1. **Was the event/incident related to the study design and / or procedure?** | | | | | **Yes** | **No** |
| 1. **Was the event/incident anticipated in the in the risks section of the approved project description?** | | | | | **Yes** | **No** |
| 1. **Will the event/incident adverse event raise additional safety concerns for the participants of this research or affect participants’ willingness to continue participation** | | | | | **Yes** | **No** |
| **Section 3: Declaration** | | | | | | |
| By submitting this form, I Chief Investigator declare that:  The information contained in this report is true and accurate. | | | | | **Yes** | **No** |

# Appendix E: Data and Safety Monitoring Board Charter

**The LifeBuoy Study: Data and Safety Monitoring Board Charter**

**Study Overview**

**The LifeBuoy App: A superiority randomised controlled trial of a mHealth intervention to help young people manage suicidal thoughts**

| Sponsor: | University of New South Wales, Sydney |
| --- | --- |
| HREC Reference number: | HC190764 |
| Clinical Trial Notification Number: | CT-2020-CTN-00256-1-v1 |
| Clinical Trials Registration Number: | ACTRN12619001671156 |
| Number of Sites: | National |
| Number of Participants: |  |

# Data and Safety Monitoring Board (DSMB) Overview

**DSMB Description**

- This DSMB members will have a degree of independence from the Black Dog Institute, University of New South Wales Sydney, and investigators, as required by the NHMRC for non-commercial trials.
- This charter will be approved by its DSMB members.
- The DSMB will function in accordance with the principles of the following documents: Good Clinical Practice (GCP) Guidelines, Declaration of Helsinki 2000, NHMRC National Statement on Ethical Conduct in Human Research, NHMRC Guidance Safety and Monitoring of Clinical Trials involving a Therapeutic Good, and University of New South Wales HREC guidelines.

**DSMB Membership Conflicts of Interest & Composition**

- Members will disclose conflicts of interest and will be cleared of significant conflicts of interest and potential conflicts of interest. No member should have financial, proprietary, professional, or other interests that may affect impartial, independent decision-making by the DSMB.
- Composition of membership will reflect exptertise in clinical trials (including a good understanding of the problems and limitations of trials), statistics and the specific scientific expertise relevant to the study, in this case, the suicidality in young people. Members will comprise 3 individuals with expertise in these areas.
- A quorum group of two must be present for closed sessions and subsequent decisions and/or recommendations made by the committee.

**Reporting**

- Data requiring review by the DSMB will be provided by the Trial Manager (Dr Lauren McGillivray) for the open session, and the data officer (Mr Adam Abedini) for the closed session.
- Issues and recommendations identified by the DSMB will be provided to Trial Steering Group (TSG), comprising the Trial Lead and Chief Investigator (Dr Michelle Tye), the Trial co-lead and Investigator (Dr Jin Han), the Trial Manager (Dr Lauren McGillivray), and the co-Chief Investigators (Dr Aliza Werner-Seidler, Dr Quincy Wong, Assoc Prof Alison Calear and Dr Bridianne O’Dea, Prof Helen Christensen) by the DSMB in accordance with this charter.

#

**Study Summary**

Involving young people aged 18 – 25 years from the Australian general community, this randomised controlled trial (RCT) will examine the use of a smartphone application (‘LifeBuoy’) to deliver digital Dialectical Behaviour Therapy (DBT) and Acceptance and Commitment Therapy (ACT) to reduce symptom severity of suicidal ideation. This study will rely on self-report data to evaluate the efficacy of the app. This study is funded by the Roth Family Foundation and an Australian Rotary Health Youth Mental Health Grant for 2 years.

**Design**

A randomised-controlled, double-blind, superiority trial with two-parallel arms (intervention and attention-matched control) with 1:1 allocation. Assessment and study procedures are automated and so the risk of differential treatment of those in the intervention and control is minimised.

**Objectives**

1. To assess the efficacy of the LifeBuoy app in reducing suicidal ideation in young people, as measured by the Suicidal Ideation Attributes Scale. Changes in suicidal ideation will be assessed by comparing the intervention and attention placebo control conditions at baseline and post-intervention, and 3-month post intervention follow-up.
2. To assess whether the LifeBuoy app significantly reduces depression and anxiety symptoms at post-intervention relative to the placebo attention control condition.
3. To examine the adherence (no. modules completed) and acceptability of the LifeBuoy app among intervention condition participants.
4. To identify predictors of change in suicidal ideation outcomes.

**Investigational Products**

**LifeBuoy.** The Lifebuoy app is a mobile app developed by researchers at the Black Dog Institute. It is a fully automated, self-help program for young people experiencing mild-to- moderate symptoms of suicidal ideation. The app has been developed using a person-centered approach, to understand and accommodate the perspectives of young people who will use the intervention. Participants will download the app from the App Store or Google Play onto their personal smartphones. Once the app is downloaded, it will not require internet connection; internet connectivity will only be required to upload usage and adherence data to UNSW servers. The app contains seven learning modules derived from Dialectical Behaviour Therapy (DBT) and Acceptance and Commitment Therapy (ACT) and incorporates wise mind principles (distress tolerance, emotion regulation/mindfulness, interpersonal effectiveness) through interactive learning exercises to help young people develop strategies and skills for managing distress. Participants have 6 weeks to complete the seven modules, each which takes approximately 3 - 5 minutes to work through. The user will be directed to each module linearly, and when an island is complete it becomes coloured to show that the user has already visited it.

**Follow-up Schedule**

All participants will complete self-report questionnaires at baseline, six-weeks post-baseline (post-intervention), and follow up. We will also conduct qualitative interviews with n=20 participants in the intervention condition at the end of the recruitment period to assess their experience of using the app. Participants will be recruited into the qualitative interviews at the start of the final assessment.

**Roles and Responsibilities**

As outlined by NHMRC Guidance on “Safety monitoring and reporting in clinical trials involving therapeutic goods” (2016), the roles of the DSMB will be to assist in:

- safeguarding the interests of study participants
- ensuring that definitive and valid results are produced which will reliably inform future healthcare decisions
- enhancing the credibility of the trial.

These roles will be achieved by examining the data accumulated during the progress of the trial, ensuring risks and benefits are monitored, and that the trial remains safe for participants. The role of the committee is to review data completeness, adverse events, outcome data (unblinded if there is good reason for this) and to recommend to the trial committee whether the study requires modification.

Recommendations will be made to the TSG, which, if appropriate, will be shared by the trial team with the Sponsor and the HREC.

**The DSMB will:**

- Following each assessment point, review aggregate subject data related to safety, attrition, withdrawals, data integrity and overall conduct of the trial and discuss via telephone conference;
- Provide recommendations to continue, modify or terminate the trial;
- Maintain records of all activities.

**The Investigators will:**

- Assure the proper conduct of the study according to the study protocol and relevant research guidelines;
- Assure collection of accurate and timely data;
- Compile and report data for the DSMB report at the end of each assessment period (trial manager and data officer);
- Monitor the project email inbox;
- Promptly report potential safety concern(s) to the DSMB;
- Communicate with regulatory authorities, e.g. HREC in a manner that maintains integrity (e.g., blinding) of the data, as necessary.

**DSMB Membership**

| Name | Affiliation | Expertise | Contact details |
| --- | --- | --- | --- |
| Prof Andrew MacKinnon | Black Dog Institute, UNSW | Clinical trial conduct; Chair | [Andrew.mackinnon@biostats.com.au](mailto:Andrew.mackinnon@biostats.com.au) |
| Prof Valsamma Eapen | School of Psychiatry, UNSW | Child and Adolescent Psychiatry | [v.eapen@unsw.edu.au](mailto:v.eapen@unsw.edu.au) |
| Dr Matthew Sunderland | Matilda Centre, University of Sydney | Statistics,  Clinical trial conduct | [matthew.sunderland@sydney.edu.au](mailto:matthew.sunderland@sydney.edu.au) |

**Selection of the Chair**

The Chair should have previous experience of serving on a DSMB and experience of chairing meetings, and should be able to facilitate and summarise the DSMB discussions.

**TSG Membership**

| Name | Affiliation | Role | Contact details |
| --- | --- | --- | --- |
| Dr Michelle Tye | Black Dog Institute, UNSW Sydney | Chief investigator, Trial Lead | [m.torok@unsw.edu.au](mailto:m.torok@unsw.edu.au) (Co-chair – open sessions) |
| Dr Jin Han | Black Dog Institute, UNSW Sydney | Investigator and co-lead | [j.han@unsw.edu.au](mailto:j.han@unsw.edu.au) (Co-chair – open sessions) |
| Dr Lauren McGillivray | Black Dog Institute, UNSW Sydney | Trial Manager | [l.mcgillivray@blackdog.org.au](mailto:l.mcgillivray@blackdog.org.au) |
| Dr Aliza Werner-Seidler | Black Dog Institute, UNSW Sydney | Investigator | [a.werner-seidler@blackdog.org.au](mailto:a.werner-seidler@blackdog.org.au) |
| Dr Quincy Wong | Western Sydney University | Investigator, statistician | [quincy.wong@wsu.edu.au](mailto:quincy.wong@wsu.edu.au) |
| Dr Bridianne O’Dea | Black Dog Institute, UNSW Sydney | Investigator | b.odea@blackdog.org.au |
| Associate Professor Alison Calear | Centre for Mental Health, ANU | Investigator | Alison.Calear@anu.edu.au |
| Professor Helen Christensen | Black Dog Institute, UNSW Sydney | Investigator | h.christensen@blackdog.org.au |

**DSMB Meetings**

The first meeting

The members should review this charter and form an understanding of the protocol and study endpoints. Meetings will take place at an appropriate regularity decided by the DSMB committee. The DSMB will also meet if evidence from other research groups suggests potential for risk.

Meeting formats

DSMB meetings will be booked in for the week following the completion of data collection at each assessment point of the trial (baseline, post-intervention, 3-month post-intervention follow-up). Procedurally, when these set meetings are booked they will include both an open and closed session, however, it is up to the DSMB to decide if they would like to have a closed session. The typical structure of the meetings will first start with an open session involving both DSMB members, the trial leads, and the trial manager, followed by the closed session if needed. The meetings will be organised and chaired by either MT or JH. These meetings will generally be conducted by teleconference or videoconference, but the DSMB can decide whether they would like to do this face-to-face.

In the event of a closed meeting, unblinded data may be requested if there is a rationale (e.g. unexpected adverse events, aggregate data indicates self-harm above the rates expected from this group). Only DSMB members and others whom they specifically invite are present in closed meetings.

Outside of the pre-scheduled meetings, an extraordinary meeting may be called if a serious adverse event occurs. The trial leads or trial manager will report the event to the Chair within 24 hours of becoming aware of the event, and the Chair will convene a closed session meeting to discuss the event and make recommendations.

Following each meeting, a report minuting the open session including any DSMB recommendations and rationale for such will be prepared by the minute taker and sent to the CIA (MT) and all other members. For closed sessions, recommendations will be prepared by the Chair and sent to the Trial Lead (MT), who will present the outcomes at the TSG following each meeting. Meetings will occur until the trial concludes.

Reporting

In terms of expedited reporting of events to the DSMB in between its regular sessions, the process will be guided by the NHMRC ‘Risk-based Management and Monitoring of Clinical Trials Involving Therapeutic Goods, 2018’, bearing in mind that this is a trial of an mhealth preventive intervention and not a medicine or treatment. The trial is regarded as being in the lowest risk category, type A, with risk comparable to standard medical care. All Serious Adverse Events (SAEs) will be regarded as non-expedited (ie. only to be reported at the next DSMB meeting), unless the SAE is both unexpected and possibly related directly to participation in the trial, in which case it will be reported to the DSMB via email within 24 hours as well as other agencies. For this 6-week low intensity app-based DBT intervention, it is anticipated that all SAEs that are possibly related to trial participation will be reported. In the event of uncertainty, the TSG should default to reporting the SAE to the DSMB.

Responsibilities of the Data Officer

The data officer will be responsible for producing the closed session report to the DSMB, and assist the trial manager with the open session report. The main purpose of this role is to ensure that the Investigator team remain blind to study outcome data. No interim analyses will be performed unless explitly required by the DSMB with a strong rational. The process for communicating the report is as follow:

- The data officer will extract unblinded data and prepare reports for the DSMB (using the attached shell) and disseminate the report one week prior to the scheduled meeting.
- The data officer will be responsible for extracting both confidential and non-confidential data from the trial data collection platform.
- The data officer will participate in the DSMB meetings to take the members through the report if required.
- These discussions will remain confidential and not communicated to the trial team.

Responsibilities of the Chief Investigator and Trial Streering Group (TSG)

- The trial leads and trial manager must be available to attend open sessions.
- Other TSG members (see membership details on p. 5) are not required to attend, but may attend if they wish to.

**Safety Analyses**

Safety analyses are:

- Suicidal ideation, as measured by:
- % scoring >20 on the SIDAS
- A recent suicide attempt (identified at post-intervention or follow up survey via Q “Have you attempted suicide in the past 30 days?’ or ad hoc self report by participants to research study email)
- Serious adverse events (including all hospitalisations and deaths).
- Enrollment data, intervention completion data, attrition data, adverse event data, and other administrative data.

The TSG cannot access the unblinded data for suicidal ideation or attempt as these are outcome measures and will be shared only in a closed session.

**Trial Stopping Guidelines**

The DSMB has the responsibility for deciding whether the trial should be stopped at any stage. They will do this if, and only if, two conditions are satisfied:

1. The results provide proof beyond reasonable doubt that the intervention is on balance definitely harmful, or for a particular category of, participants in terms of the major outcome; or is on balance, definitely having a therapeutic effect (noting that this is extremely difficult to assess without long-term follow-up periods).
2. The TSG are not conducting the project safely, which would include, but is not limited to, a failure to contact participants who flag as ‘at risk’ (scores >20 on the SIDAS) within the 48 hour period specified in the Duty of Care section within the Trial Protocol.

There is no plan to conduct any interim analyses, unless in cases where there is justification to unblind the data and condition (1) above is met. If this occurs, this DSMB Charter is in agreement with the Peto-Haybittle stopping rule whereby an interim analysis of a major endpoint would generally need to involve a difference between treatment and control of at least three standard errors to justify premature disclosure. An interim subgroup analysis would have to be even more extreme to justify disclosure. This rule has the advantage that the exact number and timing of interim analyses need not be pre-specified. In summary, the stopping rules require extreme differences to justify premature disclosure and involve an appropriate combination of mathematical stopping rules and scientific judgment.

#

**DSMB Documentation and Communication**

1. Available information at open sessions

This will include routine study information (consent, completion, attrition), risk alerts, follow-up data, by group.

1. Available information at closed sessions

Unblinded data for SIDAS and suicide attempt data and statistical analyses for this data (including effect size estimates).

1. Blinding

If necessary, the DSMB will be provided unblinded data by the data officer.

1. Access to unblinded data

Only the DSMB members, trial leads and the trial manager have access to this data. The DSMB cannot share this beyond the committee to the trial team.

1. Who is responsible for circulating external evidence (e.g., from other trials, population-based

prevalence estimates)?

Trial manager, Lauren McGillivray.

1. To whom will the DSMB communicate decisions and recommendations?

Within two weeks following each meeting, the DSMB will send findings and recommendations to the Trial Steering Group (TSG), listed on p. 5.

1. How are responses communicated back to the DSMB?

The TSG (Co-chaired by MT or JH) will review and respond to the DSMB recommendations if required. If the recommendations request action, the PI will provide a written response stating whether the recommendations will be followed and the plan for addressing the issues. Upon receipt, the DSMB will consider the TSG response and will attempt to resolve relevant issues, resulting in a final decision. The investigator will agree to disseminate the final decision to the appropriate regulatory agencies (TGA and HREC) within an appropriate time.

1. What happens to the reports after each DSMB meeting?

All DSMB members are asked to destroy reports after each meeting. Internal records will be kept by the data manager. Each report will add a table onto the previous one, and as such the reports will be cumulative.

1. What happens to minutes and records?

Any minutes should be kept by the Chairs until after the final trial analysis is complete, upon which point they should be shared with the Trial Manager for archiving for a minimum of 7 years.

**Charter agreement**

This charter was agreed by all members of the DSMB:

Signed:

Signed:

Signed:

**Date agreed:**

**Clinical Trial Protocol**

**Physiological, Psychological, Psychiatric, Surgical or Health Interventions**

The LifeBuoy App: A randomised controlled trial of a mHealth intervention to help young people manage suicidal thoughts

# Summary of Changes from Version 1.0

Dr Michelle Tye, UNSW Coordinating Principal Investigator

Version 1.1 Modifications – Approved 14.03.2020

1. **Timing of final assessment**

The final assessment timepoint was changed from 4-months post-intervention to 3-months post-intervention to accommodate delays in the development of the LifeBuoy application.

**2. Changed the minimum eligible age of participants**

The minimum age for inclusion was changed from 16 year to 18 years.

**3. Changed wording on the PISCFs**

Wording was changing on the PISCFs to improve readability and to be consistent with changes to the timelines of assessment. The major changes to the PICF are pronoun changes, re-wording the form from the original third person perspective (‘participants’, ‘young person’) to a second person perspective (‘you’) to improve the readability of the form. The timing of the final assessment was updated to accurately reflect that it would be delivered at 3-months post-intervention.

Version 1.2 Modifications – Approved 20.04.2020

**1. Addition of new tertiary and ‘other risk’ measures**

**1.1. An additional two items relating to employment status will be included in the demographic section of the baseline questionnaire. We have added the following questions to the demographic section of the baseline questionnaire:**

1. What is your current employment status? (full-time| part-time| casual| self-employed| unemployed).
2. Has your current employment status changed as a result of the coronavirus (COVID-19) pandemic? (yes| no).

**1.2. COVID-19 related worry.** Ten items are used to assess the extent to which the coronavirus (COVID-19) pandemic influences participants’ perception of their symptoms (anxiety, depression, suicidal ideation) and coping strategies. The questions are as follows:

| Do you think the COVID-19 pandemic has increased your anxiety levels more than usual? |
| --- |
| Do you think the COVID-19 pandemic has increased your depression levels more than usual? |
| If you have had recent suicidal thoughts, do you think the COVID-19 pandemic has increased the frequency or severity of these thoughts? |
| If you were to develop flu-like symptoms tomorrow, would you be worried? |
| In the past one week, have you ever worried about catching COVID-19? |
| Please rate the current level of your worry towards COVID-19 |
| *(If Q6 response of 1 or 2 then skip to Q8)* What specific aspects of COVID-19 are worrying you? |
| Think about the strategies you normally use to feel better when you’re distressed*.* Do you think you have been using these strategies more, the same, or less since COVID-19? |
| In general, do you think these strategies have been less effective during COVID-19? |
| Have you noticed any symptoms improve during, or following, the COVID-19 pandemic? |

Items 1-3 relate to perceptions of symptoms and are rated on a five-point scale, ranging from ‘Not at all (1)’ to ‘All of the time (5)’ (e.g., ‘Do you think the COVID-19 pandemic has increased your anxiety levels more than usual’); items 4-6 relate to anticipated, experienced, and current worry and were modified from a previous study of pandemic related worry, with item 7 asking participants to indicate what aspects of COVID-19 are worrying them from a list of responses (e.g., ‘not knowing when the pandemic will end’); items 8-9 relate to the frequency and effectiveness of coping strategies used, from a list of 14 equally balanced healthy (e.g., ‘using social support’ and ‘relaxation techniques’) and unhealthy (e.g., ‘over-eating/comfort food’ and ‘avoidance/procrastination’) strategies. Participants are asked to rate their use of the strategies on a four-point scale (do not use, use less, use the same, and use more), and rate on a five-point scale whether they think each strategy used has been less effective during the pandemic, from ‘Not at all (1)’ to ‘All of the time (5)’. Item 10 asks participants to indicate if they have noticed any symptoms improve during or following the pandemic (none, yes - anxiety, yes – depression, yes – suicidal thoughts, yes – other).

**1.3. Uptake of the application.** The number of persons who downloaded the application they were allocated.
