# Supplementary material for: The effect of a therapeutic smartphone application on suicidal ideation in young adults: Findings from a randomized controlled trial in Australia
Source: PLoS Med. 2022 May 31;19(5):e1003978. doi: 10.1371/journal.pmed.1003978 (PMC9154190; doi:10.1371/journal.pmed.1003978)
Supplement: S1 Table — T1, postintervention; T2, 3-month postintervention. (DOCX) [file pmed.1003978.s005.docx]

**S1 Table. Baseline characteristics of the sample for those who did or did not complete the T1 and T2 surveys**

|  | **T1 Survey** | | **T2 Survey** | | **Significance** | |
| --- | --- | --- | --- | --- | --- | --- |
|  | Completed (n=332) | Not completed (n=123) | Completed  (n=254) | Not completed  (n=201) | T1 comparisons | T2 comparisons |
| Female (n, %) | 278 (83.7) | 106 (86.2) | 214 (84.3) | 170 (84.6) | p=.66 | p=.90 |
| Age (M, SD) | 21.58 (2.18) | 21.33 (2.10) | 21.69 (2.20) | 21.29 (2.14) | p=.28 | p=.052 |
| LGBQI sexual minority, yes (n, %) | 161 (48.5) | 65 (52.8) | 122 (48.0) | 104 (51.7) | p=.45 | p=.44 |
| Education |  | | | | | |
| *Year 12 (including equivalent) or less* | 149 (44.9) | 57 (46.3) | 107 (42.1) | 99 (49.3) | p=.67 | p=.13 |
| *Graduate certificate or diploma* | 74 (22.3) | 35 (28.5) | 57 (22.4) | 52 (25.9) | p=.17 | p=.38 |
| *University Degree* | 107 (32.2) | 28 (22.8) | 88 (34.6) | 47 (23.4) | p=.06 | p=.01 |
| Not currently working/in paid employment (n, %) | 123 (37.0) | 44 (35.8) | 93 (36.6) | 74 (36.8) | p=.83 | p=1.00 |
| Ever diagnosed with a mental illness, yes (n %) | 294 (88.6) | 109 (88.6) | 218 (85.8) | 185 (92.0) | p=.87 | p=.07 |
| Have ever received mental health treatment, yes (n, %) | 296 (89.2) | 105 (85.4) | 221 (87.0) | 180 (89.6) | p=.25 | p=.56 |
| Lifetime suicide attempt, yes (n, %)^a^ | 144 (43.4) | 54 (43.9) | 114 (44.9) | 84 (41.8) | p=1.00 | p=.56 |
| SIDAS, M (SD) | 22.40 (8.34) | 23.16 (7.73) | 22.45 (8.38) | 22.81 (7.93) | p=.38 | p=.64 |
| PHQ-9, M (SD) | 16.95 (5.49) | 17.69 (6.01) | 16.55 (5.53) | 17.91 (5.70) | p=.22 | p=.01 |
| GAD-7, M (SD) | 11.96 (4.99) | 13.19 (5.19) | 11.73 (5.08) | 13.02 (4.97) | p=.02 | p=.01 |
| DQ5, M (SD) | 17.70 (3.32) | 18.37 (3.65) | 17.54 (3.34) | 18.30 (3.49) | p=.07 | p=.02 |
| SWEMWBS, M (SD) | 17.17 (2.51) | 17.09 (2.61) | 17.42 (2.44) | 16.79 (2.61) | p=.77 | p=.01 |

T1=postintervention; T2=3-months post-intervention; LGBQI=Lesbian, Gay, Bisexual, Queer, Intersex; M=Mean; SD=Standard Deviation; n=number; SIDAS =Suicidal Ideation Attributes Scale; PHQ-9=Patient Health Questionnaire -9; GAD-7=Generalised Anxiety Disorder-7; DQ5=Distress Questionnaire 5; SWEMWBS=Short Warwick–Edinburgh Mental Well-Being Scale.
