# Supplementary material for: The effect of a therapeutic smartphone application on suicidal ideation in young adults: Findings from a randomized controlled trial in Australia
Source: PLoS Med. 2022 May 31;19(5):e1003978. doi: 10.1371/journal.pmed.1003978 (PMC9154190; doi:10.1371/journal.pmed.1003978)
Supplement: S1 Text — (DOCX) [file pmed.1003978.s006.docx]

**S1 Text**

**Exploratory Analysis of Clinically Significant Change on Suicidal Ideation**

Given the clinical significance of item 5 of the SIDAS which specifically asks about life interference from suicidal ideation, we repeated the previous analyses with this item as the outcome as an exploratory exercise. There was a reduction in life interference from suicidal ideation from T0 to T1 for LifeBuoy (*B* = -1.56, 95% CI [-2.08, -1.03], t[499.02] = -5.83, p < .001, d = -0.58) but not for the control condition (*B* = -0.28, 95% CI [-0.81, 0.25], t[504.48] = -1.04, p = .301, d = -0.10), resulting in less life interference from suicidal ideation at T1 for individuals receiving LifeBuoy (*B* = -1.15, 95% CI [-1.73, -0.57], t[448.11] = -3.87, p < .001, d = 0.45). There was no significant change in life interference from suicidal ideation for LifeBuoy from T1 to T2 (*B* = -0.34, 95% CI [-0.84, 0.16], t[238.71] = -1.32, p = .187, d = -0.12), but there was a decrease in life interference from suicidal ideation for the control condition from T1 to T2 (*B* = -0.89, 95% CI [-1.40, -0.39], t[240.98] = -3.50, p = .001, d = -0.38). As a result, the lower life interference from suicidal ideation for LifeBuoy compared to the control condition observed at T1 was no longer significant at T2 (*B* = -0.59, 95% CI [-1.27, 0.09], t[494.77] = -1.71, p = .089, d = 0.23).

Table. Mean scores for the SIDAS item 5, tests of time × condition interactions, and comparisons between timepoints and conditions

|  | **LifeBuoy-C** | | | | **LifeBuoy** | | | |  | | |
| --- | --- | --- | --- | --- | --- | --- | --- | --- | --- | --- | --- |
|  | **T0**  **(M, SD)** | **T1**  **(M, SD)** | **T2**  **(M, SD)** | **Changes between timepoints^a^** | **T0**  **(M, SD)** | **T1**  **(M, SD)** | **T2**  **(M, SD)** | **Changes between timepoints^a^** | **Difference between arms at T1^a^** | **Difference between arms at T2^a^** | **Time x Condition interactions** |
| SIDAS item 5 | 4.45 (2.80) | 4.16 (2.34) | 3.43 (2.32) | ΔT0 to T1 *p* = .301  ΔT1 to T2 ***p* = .001**  ΔT0 to T2 ***p* < .001** | 4.58 (2.67) | 3.03 (2.79) | 2.84 (2.81) | ΔT0 to T1 ***p* < .001**  ΔT1 to T2 *p* = .187  ΔT0 to T2 ***p* < .001** | ***p* < .001** | *p* = .089 | T0 to T1: *B* = -1.28, 95% CI [-2.02, -0.53], *t*(501.78) = -3.37, ***p* = .001**  T1 to T2: *B* = 0.56, 95% CI [-0.15, 1.27], *t*(239.85) = 1.55, *p* = .122  T0 to T2: *B* = -0.72, 95% CI [-1.54, 0.10], *t*(584.48) = -1.72, *p* = .086 |

T0=Baseline; T1 = post-intervention; T2 = 3-month post intervention; SIDAS =Suicidal Ideation Attributes Scale. ^a^ Tests for a specific treatment arm or at a specific timepoint were conducted by recoding relevant variables representing Time and Condition within models.
